# Supplementary material for: Kernel composition in sorghum landraces revealed via analyses of genotype-by-environment interactions
Source: PLoS One. 2025 Apr 7;20(4):e0320513. doi: 10.1371/journal.pone.0320513 (PMC11975078; doi:10.1371/journal.pone.0320513)
Supplement: S1 Table — (DOCX) [file pone.0320513.s001.docx]

| **Year** | **Month** | **Melkassa** | | | | **Jimma** | | | | **Miesso** | | | |
| --- | --- | --- | --- | --- | --- | --- | --- | --- | --- | --- | --- | --- | --- |
|  |  | **Rainfall (mm)** | **Temperature ^o^C** | | | **Rainfall (mm)** | **Temperature ^o^C** | | | **Rainfall (mm)** | **Temperature ^o^C** | | |
|  |  |  | **Minimum** | **Maximum** | **Mean** |  | **Minimum** | **Maximum** | **Mean** |  | **Minimum** | **Maximum** | **Mean** |
| 2020 | January | 0.20 | 12.40 | 27.10 | 19.70 | 28.70 | 9.50 | 27.30 | 18.40 | 0.00 | 13.80 | 28.00 | 20.90 |
| 2020 | February | 0.00 | 15.00 | 30.20 | 22.60 | 38.40 | 9.50 | 28.90 | 19.20 | 1.20 | 14.80 | 30.40 | 22.60 |
| 2020 | March | 83.00 | 16.10 | 32.20 | 24.20 | 35.00 | 8.90 | 29.30 | 19.10 | 5.70 | 17.40 | 32.50 | 24.95 |
| 2020 | April | 84.40 | 16.30 | 31.00 | 23.70 | 33.70 | 10.60 | 28.00 | 19.30 | 176.60 | 18.50 | 30.60 | 24.55 |
| 2020 | May | 45.90 | 16.40 | 30.80 | 23.60 | 105.60 | 9.70 | 26.90 | 18.30 | 83.10 | 18.40 | 32.00 | 25.20 |
| 2020 | June | 183.50 | 17.40 | 29.90 | 23.70 | 154.20 | 10.30 | 28.00 | 19.10 | 44.30 | 18.90 | 33.70 | 26.30 |
| 2020 | July | 327.70 | 15.60 | 24.70 | 20.10 | 119.40 | 10.00 | 26.90 | 18.50 | 225.50 | 18.00 | 29.70 | 23.85 |
| 2020 | August | 304.50 | 15.00 | 23.70 | 19.40 | 150.90 | 10.40 | 26.50 | 18.50 | 198.60 | 18.80 | 29.30 | 24.05 |
| 2020 | September | 54.40 | 16.00 | 27.60 | 21.80 | 162.60 | 9.90 | 25.90 | 17.90 | 69.50 | 16.50 | 29.90 | 23.20 |
| 2020 | October | 1.50 | 10.70 | 29.70 | 20.20 | 210.20 | 10.10 | 27.30 | 18.70 | 6.00 | 12.70 | 31.80 | 22.25 |
| 2020 | November | 0.00 | 10.90 | 28.80 | 19.90 | 217.30 | 10.30 | 28.70 | 19.50 | 14.50 | 12.20 | 30.10 | 21.15 |
| 2020 | December | 0.00 | 9.40 | 28.20 | 18.80 | 273.80 | 9.80 | 27.50 | 18.70 | 0.70 | 10.80 | 29.00 | 19.90 |
| 2021 | January | 0.00 | 9.90 | 28.40 | 19.20 | 349.20 | 9.80 | 26.80 | 18.30 | 0.00 | 9.70 | 27.90 | 18.80 |
| 2021 | February | 33.70 | 12.30 | 28.50 | 20.40 | 115.40 | 10.60 | 27.50 | 19.10 | 0.00 | 12.80 | 29.60 | 21.20 |
| 2021 | March | 0.00 | 15.50 | 32.60 | 24.00 | 38.70 | 10.40 | 28.00 | 19.20 | 9.80 | 14.30 | 32.70 | 23.50 |
| 2021 | April | 76.20 | 15.70 | 32.40 | 24.10 | 3.30 | 10.70 | 28.30 | 19.50 | 101.80 | 17.20 | 32.60 | 24.90 |
| 2021 | May | 28.30 | 16.70 | 31.40 | 24.10 | 173.50 | 11.00 | 27.80 | 19.40 | 135.90 | 17.40 | 31.20 | 24.30 |
| 2021 | June | 40.20 | 17.10 | 32.50 | 24.80 | 179.00 | 12.10 | 28.30 | 20.20 | 0.00 | 18.70 | 34.00 | 26.35 |
| 2021 | July | 91.50 | 16.40 | 26.00 | 21.20 | 300.20 | 11.90 | 27.50 | 19.70 | 163.80 | 18.40 | 28.80 | 23.60 |
| 2021 | August | 238.60 | 15.90 | 27.80 | 21.90 | 182.20 | 11.80 | 26.70 | 19.30 | 95.60 | 17.80 | 29.90 | 23.85 |
| 2021 | September | 69.40 | 15.40 | 27.60 | 21.50 | 316.00 | 11.50 | 26.00 | 18.70 | 69.90 | 17.00 | 29.50 | 23.25 |
| 2021 | October | 51.70 | 12.00 | 28.60 | 20.30 | 169.10 | 11.10 | 26.10 | 18.60 | 51.90 | 13.80 | 30.60 | 22.20 |
| 2021 | November | 12.40 | 10.60 | 29.40 | 20.00 | 266.80 | 10.40 | 27.40 | 18.90 | 0.00 | 10.70 | 30.80 | 20.75 |
| 2021 | December | 0.00 | 10.00 | 28.60 | 19.30 | 166.80 | 10.70 | 28.30 | 19.50 | - | - | - | - |
