# Supplementary material for: Kernel composition in sorghum landraces revealed via analyses of genotype-by-environment interactions
Source: PLoS One. 2025 Apr 7;20(4):e0320513. doi: 10.1371/journal.pone.0320513 (PMC11975078; doi:10.1371/journal.pone.0320513)
Supplement: Attachment — Submitted filename: PONE-D-24-44523 revised.pdf [file pone.0320513.s003.pdf]

PLOS ONE

Kernel compositions in sorghum landraces revealed via the analyses of genotype-by-environment interactions

--Manuscript Draft--

Manuscript Number:	PONE-D-24-44523
Article Type:	Research Article
Full Title:	Kernel compositions in sorghum landraces revealed via the analyses of genotype-by-environment interactions
Short Title:	Kernel compositions in sorghum landraces revealed via the analyses of genotype-by-environment interactions
Corresponding Author:	Chalachew Endalamaw<br>Ethiopian Agricultural Research Organization: Ethiopian Institute of Agricultural Research<br>Adama, ETHIOPIA
Keywords:	sorghum; environment; GGE; grain quality; stability
Abstract:	Sorghum stands out among cereals due to its rich bioactive compounds and resilience to varying climates, addressing common issues such as low grain protein, iron (Fe), and zinc (Zn) concentration. A total of 361 sorghum landraces and four commercial checks were grown for two consecutive seasons from 2020 to 2021 at Melkassa (MK20 and MK21), Jimma (JM20 and JM21) and Miesso (MS20 and MS21). Using genotype main effects with genotype by environment interaction (GGE) ranking biplots, stable and high-performing genotypes were identified. Notably, genotypes like G246 and G84 exhibited the highest stability for starch content. For protein content, G358, G218, G221, G161, and G171 were noted for their highest mean contents and stability. For Fe content, G137 and G142 had the highest mean and were stable. For Zn, G264, G148, and G175 had the highest mean contents and were stable, while G245 and G135 displayed lower stability. MK21 emerged as an ideal environment for starch, while MS20 proved representative of protein. For Fe, environments like MS21, MK20, and MK21 were representative, while MS20 and JM20 were discriminatory. MS21 was identified as the most representative for Zn. These findings underline the diverse and specific performance of sorghum genotypes across various environmental conditions and traits.
Order of Authors:	Chalachew Endalamaw
	Dagmawit Tsegaye
	Angeline van Biljon
	Liezel Herselman
	Maryke Labuschagne
Additional Information:	
Question	Response
Financial Disclosure<br><br>Enter a financial disclosure statement that describes the sources of funding for the work included in this submission. Review the submission guidelines for detailed requirements. View published research articles from *PLOS ONE* for specific examples.	The author(s) received no specific funding for this work.

Powered by Editorial Manager® and ProduXion Manager® from Aries Systems Corporation

This statement is required for submission and **will appear in the published article** if the submission is accepted. Please make sure it is accurate.<br><br>**Funded studies**<br>Enter a statement with the following details:<br>• Initials of the authors who received each award<br>• Grant numbers awarded to each author<br>• The full name of each funder<br>• URL of each funder website<br>• Did the sponsors or funders play any role in the study design, data collection and analysis, decision to publish, or preparation of the manuscript?<br><br>Did you receive funding for this work?	
**Competing Interests**<br><br>Use the instructions below to enter a competing interest statement for this submission. On behalf of all authors, disclose any competing interests that could be perceived to bias this work—acknowledging all financial support and any other relevant financial or non-financial competing interests.<br><br>This statement is required for submission and **will appear in the published article** if the submission is accepted. Please make sure it is accurate and that any funding sources listed in your Funding Information later in the submission form are also declared in your Financial Disclosure statement.<br><br>View published research articles from *PLOS ONE* for specific examples.	The authors have declared that no competing interests exist.

**NO authors have competing interests**<br><br>Enter: *The authors have declared that no competing interests exist.*<br><br>**Authors with competing interests**<br><br>Enter competing interest details beginning with this statement:<br><br>*I have read the journal's policy and the authors of this manuscript have the following competing interests: [insert competing interests here]*<br><br>* typeset	
**Ethics Statement**<br><br>Enter an ethics statement for this submission. This statement is required if the study involved:<br><br>• Human participants<br>• Human specimens or tissue<br>• Vertebrate animals or cephalopods<br>• Vertebrate embryos or tissues<br>• Field research<br><br>Write "N/A" if the submission does not require an ethics statement.<br><br>General guidance is provided below. Consult the submission guidelines for detailed instructions. **Make sure that all information entered here is included in the Methods section of the manuscript.**	study involving 361 genotypes over two seasons at two locations at EIAR, you might consider the specific focus of your research, such as environmental impact, genotype performance, or trait association

Powered by Editorial Manager® and ProduXion Manager® from Aries Systems Corporation

**Format for specific study types**<br><br>**Human Subject Research (involving human participants and/or tissue)**<br>• Give the name of the institutional review board or ethics committee that approved the study<br>• Include the approval number and/or a statement indicating approval of this research<br>• Indicate the form of consent obtained (written/oral) or the reason that consent was not obtained (e.g. the data were analyzed anonymously)<br><br>**Animal Research (involving vertebrate animals, embryos or tissues)**<br>• Provide the name of the Institutional Animal Care and Use Committee (IACUC) or other relevant ethics board that reviewed the study protocol, and indicate whether they approved this research or granted a formal waiver of ethical approval<br>• Include an approval number if one was obtained<br>• If the study involved *non-human primates*, add *additional details* about animal welfare and steps taken to ameliorate suffering<br>• If anesthesia, euthanasia, or any kind of animal sacrifice is part of the study, include briefly which substances and/or methods were applied<br><br>**Field Research**<br><br>Include the following details if this study involves the collection of plant, animal, or other materials from a natural setting:<br>• Field permit number<br>• Name of the institution or relevant body that granted permission	
**Data Availability**<br><br>Authors are required to make all data underlying the findings described fully available, without restriction, and from the time of publication. PLOS allows rare exceptions to address legal and ethical concerns. See the PLOS Data Policy and FAQ for detailed information.	Yes - all data are fully available without restriction

A Data Availability Statement describing where the data can be found is required at submission. Your answers to this question constitute the Data Availability Statement and **will be published in the article**, if accepted.<br><br>**Important:** Stating 'data available on request from the author' is not sufficient. If your data are only available upon request, select 'No' for the first question and explain your exceptional situation in the text box.<br><br>Do the authors confirm that all data underlying the findings described in their manuscript are fully available without restriction?	
**Describe where the data may be found in full sentences. If you are copying our sample text, replace any instances of XXX with the appropriate details.**<br><br>• If the data are **held or will be held in a public repository**, include URLs, accession numbers or DOIs. If this information will only be available after acceptance, indicate this by ticking the box below. For example: *All XXX files are available from the XXX database (accession number(s) XXX, XXX.*).<br>• If the data are all contained **within the manuscript and/or Supporting Information files**, enter the following: *All relevant data are within the manuscript and its Supporting Information files.*<br>• If neither of these applies but you are able to provide **details of access elsewhere**, with or without limitations, please do so. For example:<br><br>*Data cannot be shared publicly because of [XXX]. Data are available from the XXX Institutional Data Access / Ethics Committee (contact via XXX) for researchers who meet the criteria for access to confidential data.*<br><br>*The data underlying the results presented in the study are available from (include the name of the third party*	No

and contact information or URL).<br>• This text is appropriate if the data are owned by a third party and authors do not have permission to share the data.<br><br>* typeset	
Additional data availability information:	Tick here if the URLs/accession numbers/DOIs will be available only after acceptance of the manuscript for publication so that we can ensure their inclusion before publication.; Tick here if your circumstances are not covered by the questions above and you need the journal's help to make your data available.

Manuscript

Click here to access/download;Manuscript;Kernel compositions in sorghum landraces.docx

Kernel compositions in sorghum landraces revealed via the analyses of genotype-by-environment interactions

Chalachew Endalamaw[1,2*], Dagmawit Tsegaye[1], Angeline van Biljon[2], Liezel Herselman[2], Maryke Labuschagne[2]

[1]Ethiopian Institute of Agricultural Research, Addis Ababa, Ethiopia
[2]Department of Plant Sciences, University of the Free State, Bloemfontein, South Africa

Abstract

Sorghum stands out among cereals due to its rich bioactive compounds and resilience to varying climates, addressing common issues such as low grain protein, iron (Fe), and zinc (Zn) concentration. A total of 361 sorghum landraces and four commercial checks were grown for two consecutive seasons from 2020 to 2021 at Melkassa (MK20 and MK21), Jimma (JM20 and JM21) and Miesso (MS20 and MS21). Using genotype main effects with genotype by environment interaction (GGE) ranking biplots, stable and high-performing genotypes were identified. Notably, genotypes like G246 and G84 exhibited the highest stability for starch content. For protein content, G358, G218, G221, G161, and G171 were noted for their highest mean contents and stability. For Fe content, G137 and G142 had the highest mean and were stable. For Zn, G264, G148, and G175 had the highest mean contents and were stable, while G245 and G135 displayed lower stability. MK21 emerged as an ideal environment for starch, while MS20 proved representative of protein. For Fe, environments like MS21, MK20, and MK21 were representative, while MS20 and JM20 were discriminatory. MS21 was identified as the most representative for Zn. These findings underline the diverse and specific performance of sorghum genotypes across various environmental conditions and traits.

Keywords: sorghum, environment, GGE, grain quality, stability

Introduction

Sorghum (*Sorghum bicolor* L. Moench) is a vital cereal crop, particularly in semi-arid regions of Africa and Asia, where it plays a crucial role in food security and nutrition [1,2]. With its inherent drought tolerance and adaptability to marginal environments, sorghum is a staple for millions of people in these areas. Variations in both physical and chemical components of the sorghum grain were observed due to the diverse set of sorghum genotypes and variations in growth conditions [3]. The physical attributes of the grain, including kernel size and hardness, displayed more variability as a result of the interaction between genotype and location [3]. Beyond providing caloric intake, sorghum is increasingly recognized for its potential to enhance the nutritional status of vulnerable populations due to its considerable variation in grain nutritional content, including protein, iron (Fe), and zinc (Zn) concentrations [4,5]. These micronutrients are essential for addressing widespread deficiencies that contribute to malnutrition and related health issues, especially in developing countries.

Despite the importance of sorghum in contributing to human nutrition, the expression of nutritional traits is highly influenced by genotype-by-environment interactions (GEI), making the identification of stable and high-performing genotypes across different environments a challenge. Understanding these interactions is critical for breeding programs that aim to improve sorghum's nutritional quality and ensure consistent performance across diverse agroecologist. The genotype main effects and genotype-by-environment interaction (GGE) biplot analysis has become a powerful tool for analyzing multi-environment trial (MET) data [6,7,8]. By integrating both genotype and GxE effects, the GGE biplot facilitates the identification of genotypes that are not only high-performing but also stable across different environments [9,10]. Furthermore, this method allows for the characterization of testing environments in terms of their representativeness and discriminatory power, aiding in the selection of optimal testing sites for future breeding efforts.

Adaptability and yield stability are important measures for crop cultivation in different agro-climatic regions. The stability and adaptability of genotypes across different environments can be assessed using various statistical tools such as joint regression [11 and genotype main effects in addition to GGE biplots [12]. GGE biplots serve as the most efficient and commonly

used multivariate models for determining genotype stability, adaptability, and ranking, as well as for identifying suitable mega-environments[7,13,14,15,16].

Most sorghum landrace studies have focused on the evaluation of grain yield, with less emphasis placed on grain quality traits using multi-environment trial (MET) data. The environmental effects on grain quality on a diverse set of sorghum genotypes have not been extensively studied. Although GEI has been used to assess the stability of improved varieties of sorghum using MET data [17,18,19], information on the stability of sorghum landraces for quality traits using GGE biplot models is lacking.

The extensive variety of sorghum cultivation areas, coupled with genetic diversity, presents considerable difficulties in ensuring consistent quality across different growing regions. Consequently, this research aims to assess how diverse genotypes and various growing locations impact the chemical and physical properties of sorghum grain. The objectives include identifying genotypes that consistently maintain quality across diverse growing locations and examining the environmental interaction with sorghum quality attributes.

Materials and methods

Study areas and crop management

This study quantified GEI for the wet intermediate (Jimma) and dry lowlands (Melkassa and Miesso) agroecology from 2020 to 2021. The two dry lowland experimental sites are found in the central rift valley of Ethiopia. Jimma is in the wet intermediate agroecology with good rainfall in the cropping season. Both Miesso and Melkassa are lowland sorghum production areas and moisture-stress sorghum testing sites in Ethiopia (Figure 1) [20]. Tables 1 to 2 and Appendix 1 show data for the three sites on planting and harvesting dates, rainfall patterns, relative humidity, maximum/minimum temperatures, and soil analytical data for macronutrients and organic matter. Seeds were drilled in the row at 75 cm between rows soon after plowing and then thinned to maintain 15 cm intra-row spacing at all sites. Basal fertilizer was applied at the recommended rate and other agronomic practices were applied for normal sorghum production in Ethiopia.

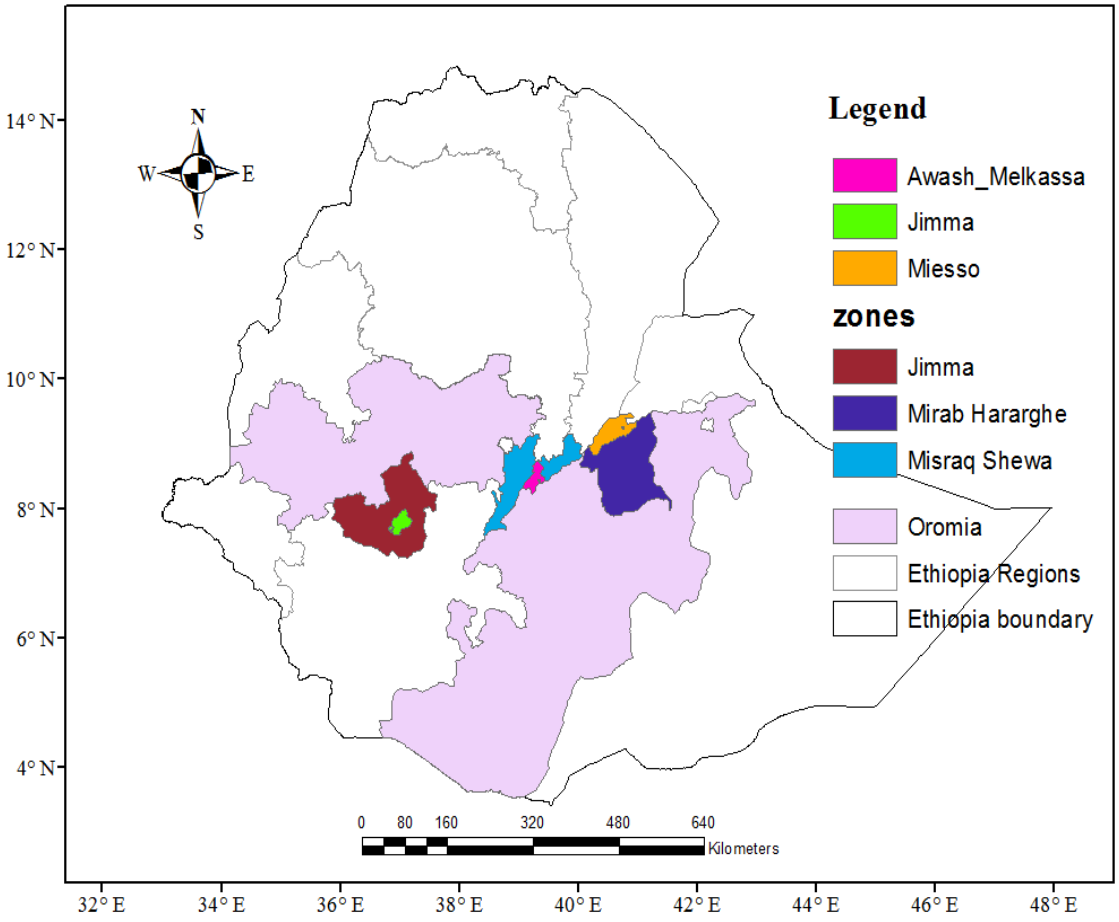

Figure 1. The three test locations in Ethiopia, Jimma, Miesso, and Melkassa, are depicted on a map using an ArcGIS geographic information system [21].

Jimma, Miesso, and Melkassa are situated in the Oromia Regional State's Jimma, West Hararghe (Mirab Hararghe), and East Hararghe (Misrak Hararghe) zones, respectively.

Table 1. Long-term average temperature and rainfall properties for each trial site

Location	Longitude	Latitude	Altitude (m.a.s.l)	Soil type	Rainfall (mm)	Minimum T°C	Maximum T°C
**Miesso**	39°21'E	8°30'N	1 470	Clay loam	570	16	31
**Melkassa**	39°9'E	14°6'N	1 550	Clay loam	763	20.4	34
**Jimma**	36°47′ E	7°40′ N	1 753	Clay	1 500	20	29

m.a.s.l. = meter above sea level; T°C = temperature in degrees centigrade.

Table 2. The planting and harvesting dates of trials in two consecutive seasons

	Planting date		Harvesting date	
**Location**	**Year 1**	**Year 2**	**Year 1**	**Year 2**
Melkassa	Jul 15, 2020	Aug 3, 2021	Jan 1-20, 2021	Jan 10-12, 2022
Miesso	Jul 20, 2020	May 11, 2021	Nov 27- Dec 5, 2020	Aug 30-Dec 24, 2021
Jimma	May 26, 2020	May 6, 2021	Dec 8, 2020 to Jan 8, 2021	Dec 4-30, 2021

Table 3. Soil analytical data for some macronutrients and organic matter for the three trial sites

Environment	Soil depth (cm)	pH	EC (µS/cm)	Textural class	%TOC	%OM	%TN	Av. P (ppm)
Jimma	0-30	6.67	96.50	Clay	1.57	2.71	0.16	8.45
	30-60	6.88	72.70	Clay	1.21	2.09	0.13	8.33
Melkassa	0-30	6.79	190.80	Clay loam	1.29	2.23	0.08	7.95
	30-60	7.28	230.00	Clay	1.25	2.16	0.08	7.73
Miesso	0-30	7.25	139.70	Clay loam	1.06	1.83	0.09	8.73
	30-60	7.28	231.50	Clay	1.03	1.78	0.09	8.61

EC = Electrical conductivity; TOC = Total organic carbon; OM = Organic matter; TN = Total nitrogen; Av. P = available phosphorus.

Design and data analysis

The 361 Ethiopian sorghum landraces and four improved varieties were evaluated for genetic diversity and GEI in two consecutive seasons (2020 to 2021).

Due to the large number of landrace landraces included in this study, partial replication was used in all trial designs [22]. This customized design was used to minimize spatial error effects within each trial and had a row-column arrangement. A multi-environment trial design with 354 landraces and four commercial checks, totaling 358 sorghum entries across three sites (Jimma, Melkassa, and Miesso) was used. Of the total of 354 sorghum landraces, a third were replicated at each site and the replicated set was different at each site. The four checks were replicated at all sites. Each plot consisted of a single row, 4 m in length. The experimental design consisted of 10 columns and 48 rows, with each row containing a different sorghum landrace. A total of 48 sorghum landraces were organized per single column. This experimental design allowed for the testing of the effects of both genotype and environment, as well as GEI. The sorghum grain quality data collected from

the six environments were subjected to a combined analysis of variance (ANOVA) using the mixed linear model in R software [23]. METs were analyzed by fitting a linear mixed model using the spatial analysis with package ASReml [24]. Each model consisted of a fixed effect for the targeted trait at each trial, random effects (BLUPs) for genotypes within the trial, and spatial error for each trial [25]. The GGE biplot analyses were conducted using GenStat software, version 15 [26].

Analysis of sorghum grain quality

The Melkassa Agricultural Research Center established a near-infrared spectroscopy (NIRS) calibration model to assess sorghum grain quality, providing a cost-effective and time-efficient alternative to the more expensive and time-consuming wet chemistry analysis. The development of NIRS models for sorghum grain quality analysis involved utilizing over 108 Ethiopian sorghum accession samples [27].

The [27] observed that the NIRS calibration equations demonstrated high coefficients of determination for calibrations ($R^2c = 0.815$) and slightly lower coefficients for cross-validations ($R^2cv = 0.589$) concerning tannin content. Similarly, the total starch content showed high $R^2c = 0.983$ with slightly lower $R^2cv = 0.910$, while amylose content exhibited high $R^2c = 0.762$ and slightly lower $R^2cv = 0.697$. The standard error of calibration (SEC) and standard error of cross-validation (SECV) were minimal (SECV/mean = 3.8%, 1.2%, and 4.3% for tannin, total starch, and amylose, respectively).

In this investigation, sorghum landrace collection samples underwent NIRS scanning at Melkassa Agricultural Research Centre. The percentage of sorghum grain protein, ash, starch, amylose, amylopectin, and moisture, as well as the parts per million (ppm) of Zn and Fe, were determined using NIRS. An Inframatic 9500 NIR System Grain Analyzer scanned 200 g of cleaned whole grain for every environment.

Results

Genotypic variation, genotype by environment interaction

Highly significant genotype (σ^2g), environment (σ^2en) and genotype x environment (σ^2gen) interaction variance effects were present for all traits (Table 4). Variations in quality traits among different genotypes were found to be significant, indicating distinct differences in the selected genotypes. The analysis of variance revealed that both genotype and environmental factors significantly influence sorghum grain quality, emphasizing the importance of considering these components in the evaluation of sorghums for potential end-use.

The starch, amylose, amylopectin, protein, Fe, Zn, ash, and moisture contents exhibited a wide variation due to genotypes across all environments (Table 4 and Appendix 2). The starch content ranged from 67.44 to 69.65 % in genotypes G355 (IS38319) and G4 (Bonsa), respectively (. The amylose content ranged from 19.04 to 20.66% in genotypes G48 (ETSL100178) and G264 (ETSL101325), respectively. The amylopectin content varied from 79.64 (G264) to 80.97 % (G48). Genotype G48 (ETSL100178) could be considered hetero-waxy sorghum due to its lower amylose and higher amylopectin values. The genotype played a major role in influencing the amylose content of the starch, with the majority of cultivated sorghums characterized by "normal" type starches containing both amylose and amylopectin. The protein content ranged from 7.69 to 11.11 % in genotypes G202 (ETSL101021) and G358 (IS38353), respectively. The Fe content ranged from 12.08 to 80.77 ppm in genotypes G6 (DS10) and G142 (ETSL100735), respectively. The Zn content ranged from 21.92 to 58.04 ppm in genotypes G296 (ETSL101492) and G264 (ETSL101325), respectively. The ash content ranged from 14.35 to 16.24% in genotypes G96 (ETSL100443) and G2649 (ETSL101325), respectively. Genotype G264 (ETSL101325) had the highest values for amylose, Zn, and ash while had the lowest for amylopectin. The moisture content ranged from 14.35 to 16.24% in genotypes G245 (ETSL101255) and G355 (IS38319), respectively. Genotype G355 (IS38319) had the highest value for moisture but had the lowest value for starch. The relationship between moisture and starch content is inverse, indicating that as moisture content increases, starch content tends to decrease.

Table 4. Genotype ($\sigma^2 g$), environment ($\sigma^2 en$), and genotype x environment ($\sigma^2 gen$) interaction variances, and standard errors (SE) for quality traits.

Trait	Range	Mean	$\sigma^2 g$	SE	$\sigma^2 gen$	SE	$\sigma^2 en$
Starch	64.44 -69.65	68.75	0.30**	0.06	0.29**	0.11	2182.00**
Amylose	19.04-20.66	19.60	0.01**	0.00	0.01**	0.00	1967.00**
Amylopectin	79.64-80.97	80.40	0.01**	0.00	0.01**	0.00	1967.00**
Protein	7.69-11.11	9.55	0.45**	0.05	0.227**	0.06	1321.00**
Fe	12.08-80.77	32.28	11984.76**	126.11	0.11**	0.32	748.21**
Zn	21.92-58.04	28.06	4.38**	0.67	4.32*	1.79	250.70*
Ash	1.22-2.98	1.81	0.09**	0.01	0.05**	0.01	537.60**
Moisture	14.35-16.24	15.50	0.12**	0.03	0.16**	0.05	2933.00**

* $P \leq 0.05$; ** $P \leq 0.01$.

Genotype main effects with genotype by environment interaction biplot analysis

"Which-won-where" polygon of GGE biplot

The GGE biplot polygon view identified the highest-performing genotypes by showing interaction patterns among genotypes and environments (Figures 5 and 6). A polygon was drawn in this GGE biplot by connecting the vertex genotypes, which were far from the origin, with straight lines, and thus all the other genotypes were enclosed within the polygon. Genotypes located at the polygon's vertices showed the best performance or worst in one or more environments. The starch vertex genotypes were G265, G84, G246, G4, G40, G110, G36, G264 and G355. The protein vertices genotypes were G358, G161, G232, G346, G346, G202, G142, G94, G207 and G98. G142, G137, G194, G311, G272, G6, G18, G325, G190 and G119 were selected for the Fe vertex. The Zn vertices were G264, G249, G296, G285, G245 and G135 (Figure 5). As a result, these four groups of genotypes were the most responsive to environmental interactions for starch, protein, Fe, and Zn, in that order.

In the "which-won-where" GGE biplot, lines from the origin divide the biplot into different sectors and create different mega environments (MGEs). In this study, all traits had two MGEs formed, except for Fe which only had one. For starch, environments JM20, MK20, MK21, MS20, and MS21 formed a joint MGE, whereas environment JM21 was a separate MGE for these traits. For protein, environments JM20, MK20, MK21, MS20, and JMS21 formed an MGE together, whereas environment MS21 was a separate MGE for this trait (Figure 5). For Zn, environments JM20, MK20, MK21, JM21, and MS21 formed a joint MGE, whereas environment MS20 formed a separate MGE for this trait, whereas for Fe, all environments formed a single MGE (Figure 6).

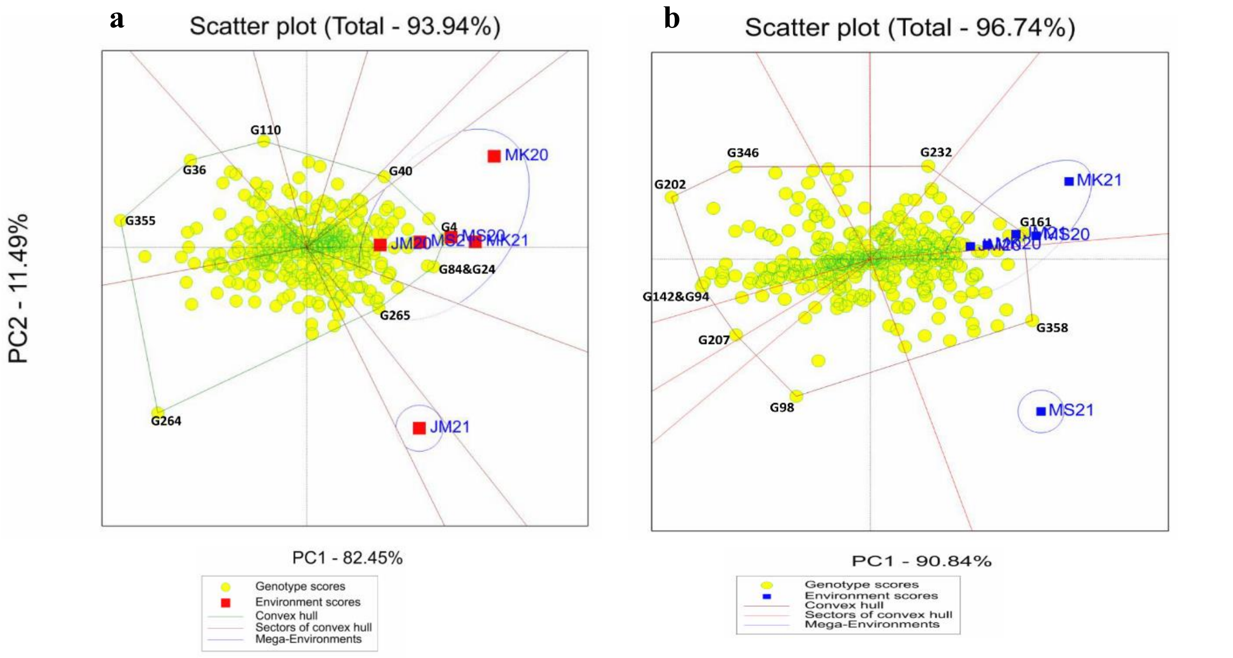

Figure 2. “Which-won-where” polygon view of genotype main effects with genotype by environment interaction scatter biplot of the 365 sorghum landraces for (**a**) starch and (**b**) protein, displaying landraces with the best performance in each environment and the mega environments. Vertex landraces on convex hulls (polygon) are the greatest for each mega environment for the correlating parameters.

JM20 and JM21 = Jimma 2020 and 2021; MK20 and MK21 = Melkassa 2020 and 2021; MS20 and MS21 = Miesso 2020 and 2021, respectively.

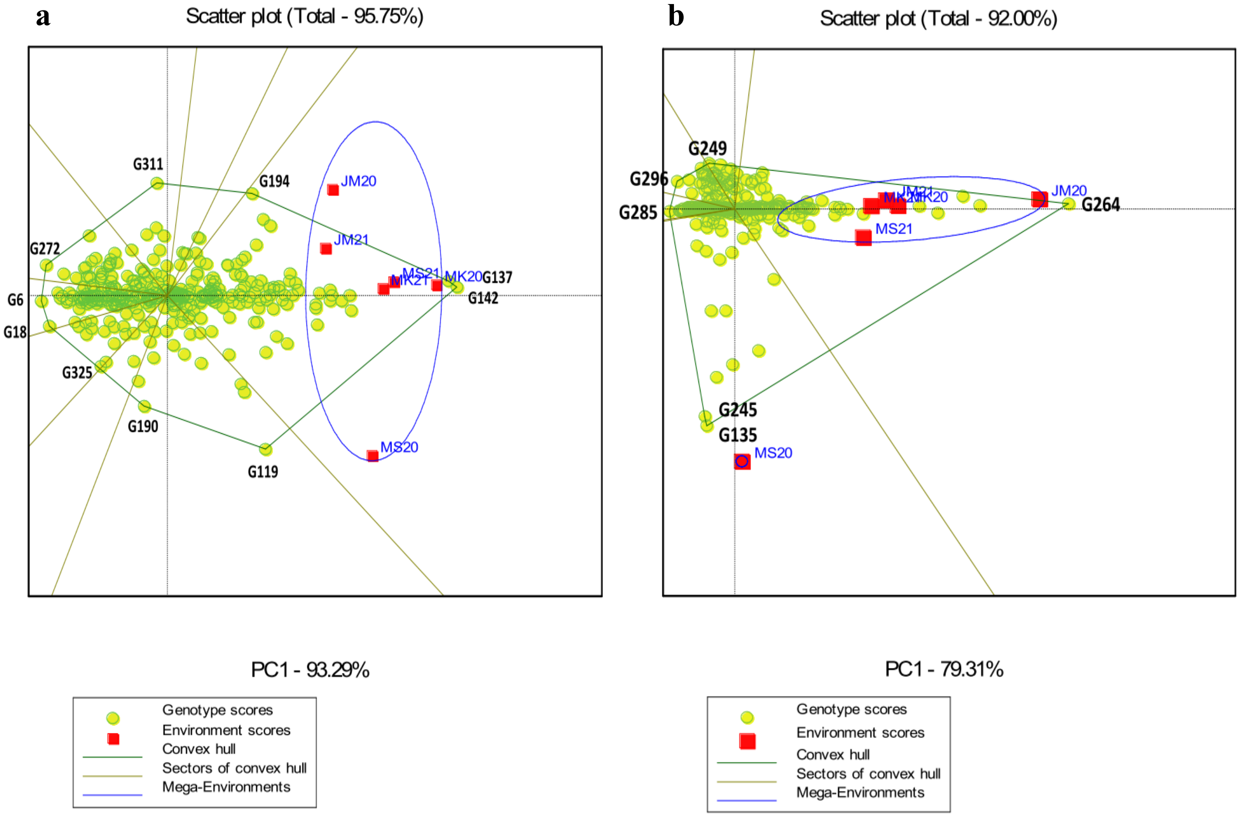

Figure 3. "Which-won-where" polygon view of genotype main effects with genotype by environment interaction scatter biplot of the 365 sorghum landraces for (**a**) Fe and (**b**) Zn, displaying landraces with the best performance in each environment and the mega environments. Vertex landraces on convex hulls (polygon) are the greatest for each mega environment for the correlating parameters.

JM20 and JM21 = Jimma 2020 and 2021; MK20 and MK21 = Melkassa 2020 and 2021; MS20 and MS21 = Miesso 2020 and 2021, respectively.

Genotypes G4 (Bonsa), G246 (ETSL101256), and G84 (ETSL100406) performed well in environments for starch concentrations. Whereas genotype G161 (ETSL100834) performed well in all environments for protein concentrations in 2020 and 2021, G358 (IS38353) performed well under MS21. Genotypes G355, G36, and G264 had low starch contents, while genotypes G142, G94, G207, G202, and G98 had low protein contents in all environments (Figure 5). Genotype G264 (ETSL101325) performed well in the control and stress conditions for Zn concentrations, whereas G245 (ETSL101255) and G135 (ETSL100798) performed well in the MS20 stress condition. For Fe concentrations, genotypes G137 (ETSL100702) and G142 (ETSL100735)

performed well in the control and stress conditions. Genotypes G272, G6, G18, and G325 had low Fe contents, while genotypes G285 and G296 had low Zn contents in all environments (Figure 6).

Genotype performance and stability

Genotypes should be assessed for average performance and stability over all environments within a single mega-environment. The single-arrowed line on a GGE biplot indicates the average environment coordinate (AEC) and indicates that the mean trait value is higher across environments. A genotype with a greater mean performance has an average environment axis (AEA) in the ranking biplot, represented by a single-arrowhead line passing through the origin. Thus, G246 and G84 had the highest mean starch contents, followed by G4, G175, and so on; G170, G35, G215, G106, and G193 had mean starch contents that were similar to the grand mean; and G355 and G212 had the lowest mean starch contents. In terms of protein, G358, G218, G161, G171 and G221 had the highest mean protein contents, followed by G43, G88, G293, G184 and so on; G170, G35, G215, G106, and G208 had mean protein contents similar to the grand mean; and G202, G63, G142 and G94, G346, and G207 had the lowest mean protein contents (Figure 7). For Fe concentrations, G137 and G142 had the highest mean Fe contents, followed by G166, G248, and so on. G88 and G162 had mean Fe contents similar to the grand mean and G272, G6, and G18 had the lowest mean Fe contents. G264 had the highest mean Zn content, followed by G142, G278, G175, G148, and so on; G119 and G90 had mean Zn contents similar to the grand mean (Figure 8).

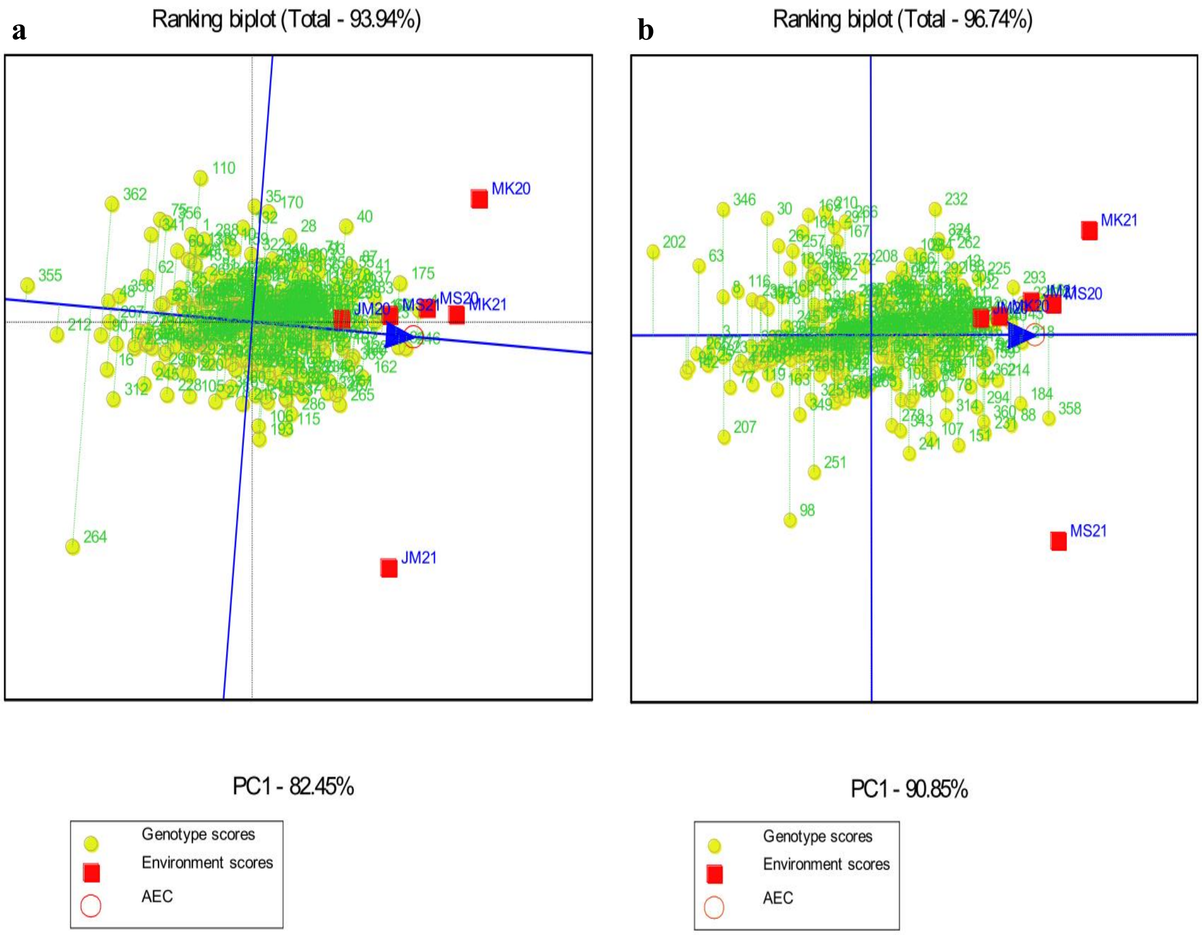

Figure 4. Genotype main effects with genotype by environment interaction biplot genotype focus scaling displaying the stability and mean priority of the 356 sorghum landraces for (**a**) starch and (**b**) protein.

JM20 and JM21 = Jimma 2020 and 2021; MK20 and MK21 = Melkassa 2020 and 2021; MS20 and MS21 = Miesso 2020 and 2021.

The stability of genotypes was assessed using the distance of the vector between genotype positions and the AEA in the ranking biplot. The best-performing and most stable genotypes are those that are far from the origin but on or near the AEA. As a result, G246 and G84 were the most stable genotypes with high mean starch contents and the shortest vector from AEA, whereas G264 and G110 were the least stable genotypes with the longest vector from AEA. The highest mean protein content and shortest AEA vectors were found in G218, while the lowest mean protein contents and longest AEA vectors were found in G210, G232, G98, G346, G207, and G251 (Figure 7).

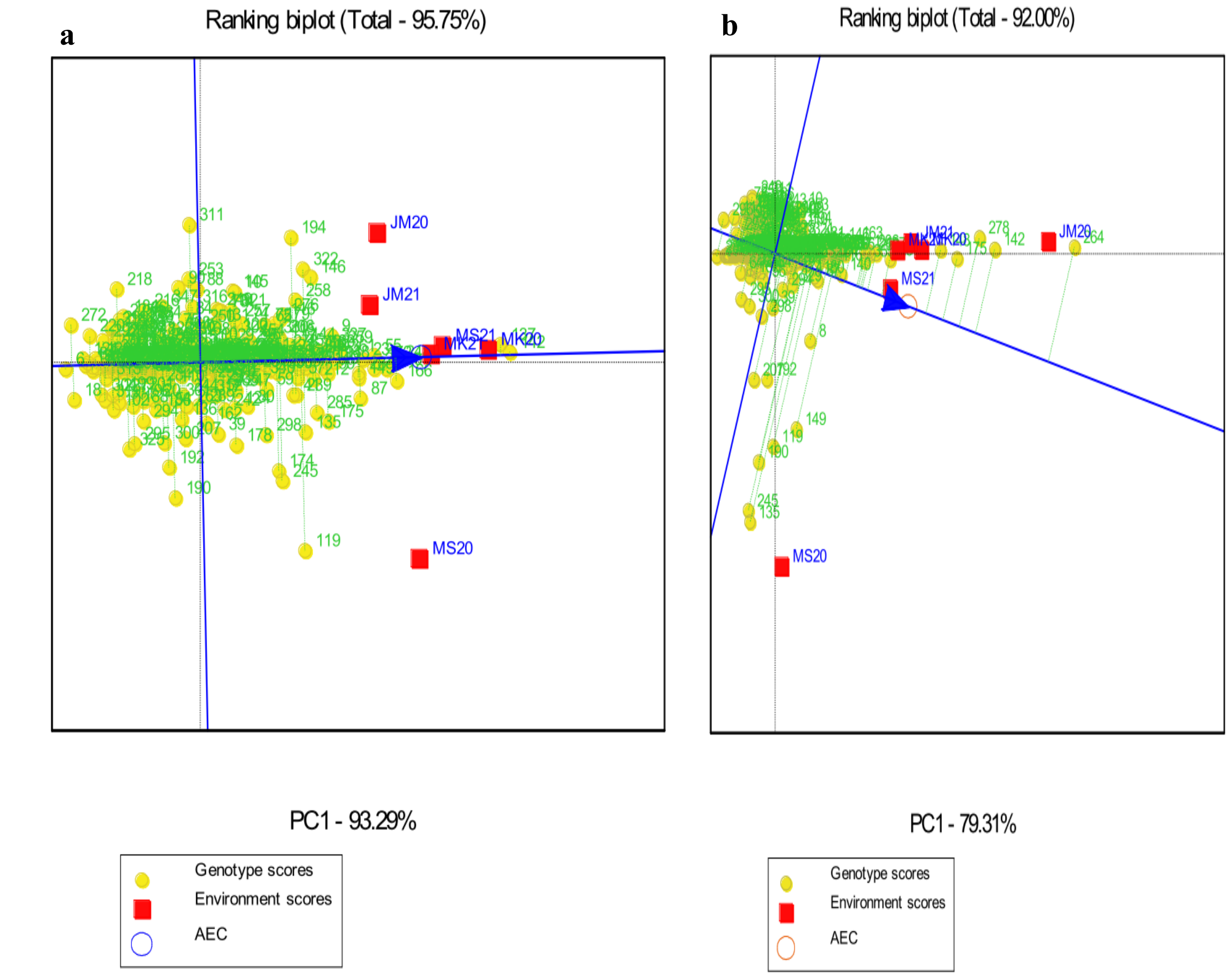

Figure 5. Genotype main effects with genotype by environment interaction focus scaling displaying the stability and mean priority of the 356 sorghum landraces for (**a**) Fe and (**b**) Zn.

JM20 and JM21 = Jimma 2020 and 2021; MK20 and MK21 = Melkassa 2020 and 2021; MS20 and MS21 = Miesso 2020 and 2021.

The highest mean Fe content and shortest AEA vectors were found in G137 and G142 and these genotypes were thus the most stable genotypes with high mean Fe contents and shorter AEA vectors, whereas G311, G194, and G119 were the least stable genotypes with the longest AEA vector. Meanwhile, G148 and G175 had the highest mean Zn contents and the shortest AEA vector, and G245 and G135 had the lowest mean Zn contents and the longest AEA vector (Figure 8).

Evaluation of the testing environment

The AEA, also known as the average-tester-axis, contains the AEC and ideal environment (shown by the circle at the end of the arrow in Figures 9 and 10) of all test environments and the AEA is the line that passes through the biplot origin and ideal environment. The GGE biplot displays AEA, AEC, ideal environment, and concentric circles to evaluate the tested environments. The ideal test environment should be both discriminating and representative of the target environments and be on or near the center of the concentric circles. A smaller angle with the AEA in a test environment is more representative of other test environments while test environments that are close to AEC are more representative of other environments.

In this study, for starch content, if the target environments are distributed into mega-environments, the discriminating but non-representative test environments MK20 and JM21 can be used to select genotypes with specific adaptations. The environment of MK21 was the closest to the ideal environment and thus the best at selecting cultivars adapted to the entire region. It should be noted that more years are required to confirm that a particular test location is ideal (Figure 9a). For protein content, MS20 was the most representative and ideal test environment, as well as the closest to the AEC, while MS21 was the most discriminating environment for protein content (Figure 9b). Environments with the highest Fe content, MS21, MK20, and MK21, were representative test environments with the smallest angles and were closest to AEC; on the other hand, MS20 and JM20 were discriminating environments for Fe concentrations (Figure 10a). The most representative environment for Zn was MS21, while the ideal and discriminating test environments were JM20 and MS20, respectively (Figure 10b).

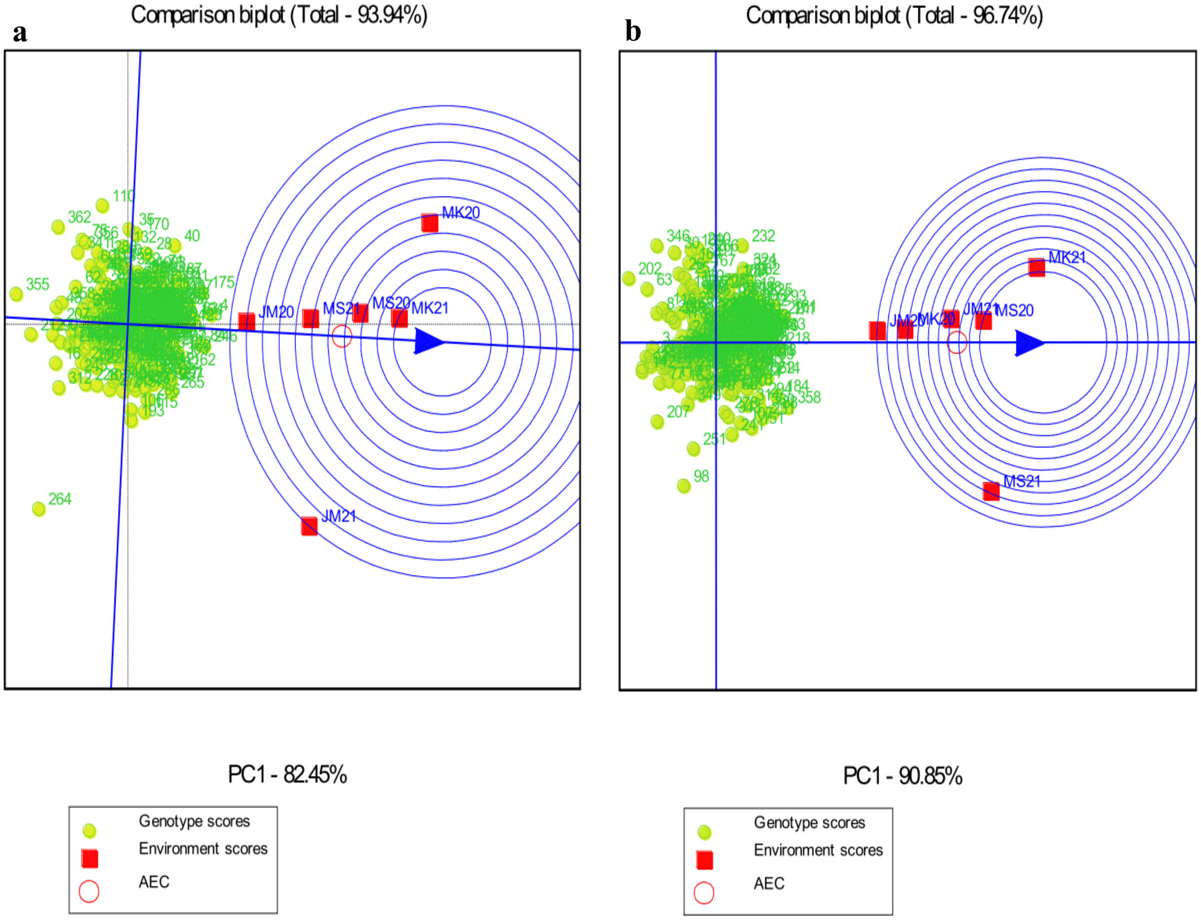

Figure 6. The discrimination and representativeness view of the genotype main effects with genotype by environment interaction biplot used to rank test environments relative to an ideal test environment (represented by the center of the concentric circles) for (**a**) starch and (**b**) protein.

JM20 and JM21 = Jimma 2020 and 2021; MK20 and MK21 = Melkassa 2020 and 2021; MS20 and MS21 = Miesso 2020 and 2021.

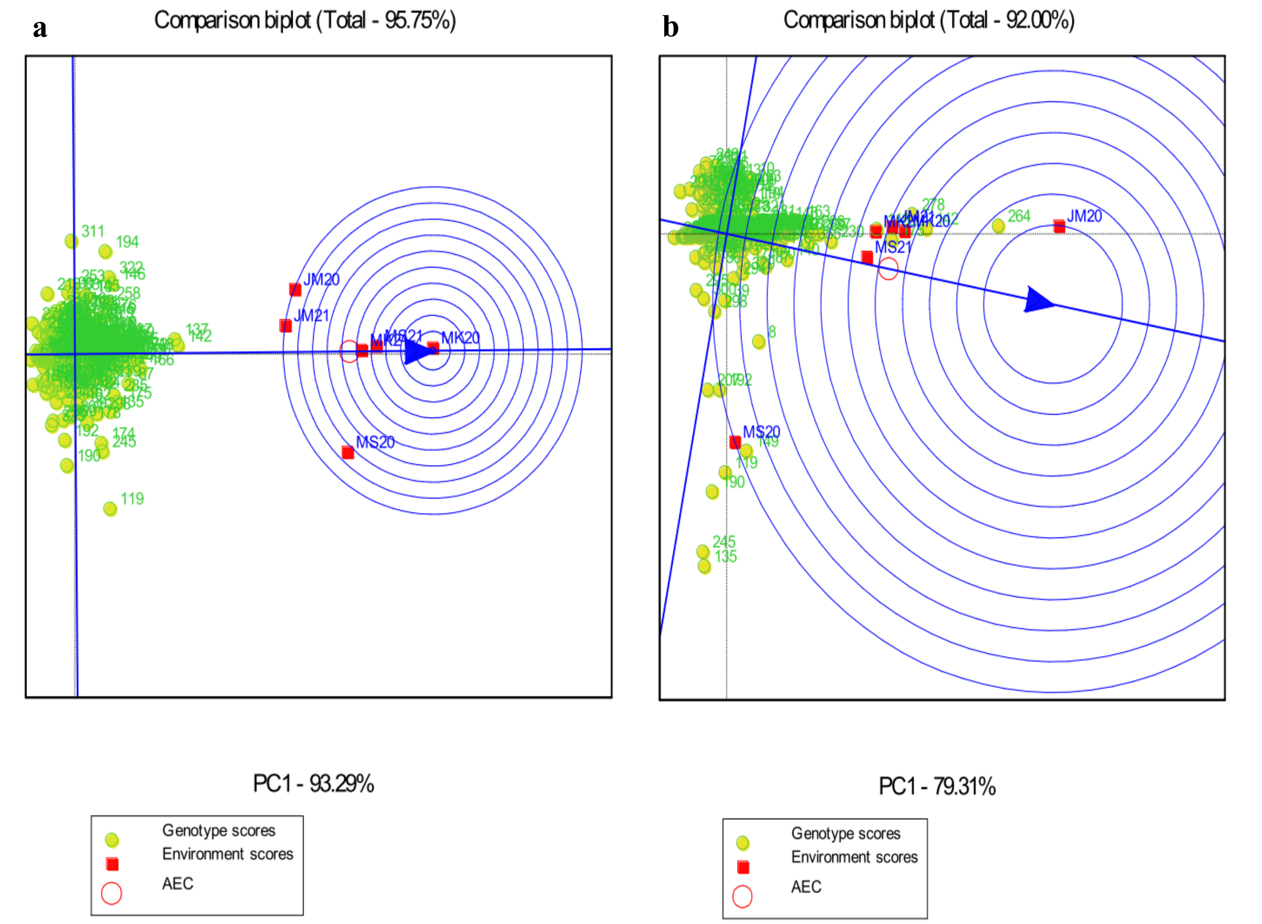

Figure 7. The discrimination and representativeness view of the genotype main effects with genotype by environment interaction biplot used to rank test environments relative to an ideal test environment (represented by the center of the concentric circles) for (**a**) Fe and (**b**) Zn.

JM20 and JM21 = Jimma 2020 and 2021; MK20 and MK21 = Melkassa 2020 and 2021; MS20 and MS21 = Miesso 2020 and 2021.

Discussion

The performance of promising genotypes must be repeatable and stable across years to identify and recommend superior genotypes. To accomplish this, it is crucial to conduct a MET on unbalanced data, incorporating factor analysis along with mixed models. This approach is vital for accurately gauging the relative stability and GEI of varieties.

The capacity to choose the most desirable trait at a specific location is facilitated by the variation attributed to genotype and location [3]. Environmental influences on starch and amylose content have been observed in other cereal grains, such as wheat [28], and rice [29]. The amylose/amylopectin ratio holds significance for various functional properties of starch, including its gelatinization profile [30]. Given the extensive genetic diversity within this sample set, the observed range in protein content was anticipated and aligned with the typical protein range found in sorghums [31].

Variation between environments was found in the comparison of GGE biplots for grain Fe and Zn [32]. Grain Zn and Fe showed significant GEI, highlighting the need for further research to address the GEI and create sorghum landraces that are high in micronutrients. Fe and Zn concentrations in grain may vary according to soil micronutrient levels. Another reason for the high GEI for Fe and Zn content could be their heritability [33,34]. The difference in the relative performance of genotypes in different environments is a strong indicator of GEI, as well as variation in environmental conditions such as temperature, rainfall, and soil type. Starch, the main macronutrient, was reported to have a strong negative correlation with crude protein when grain macronutrients were measured on a percent dry matter basis [35]. Fe and Zn contents had a significant positive relationship over environments, indicating the possibility of concurrently selecting for both traits [32], which was also the case in the current study.

The GGE “which-won-where” biplot was applied to distinguish top-performing genotypes by interpreting GEI, MGE clustering, and specific adaptation [16,36,37]. The unstable genotypes are those that are far away from the biplot origin (vertex genotypes) and perform best or worst in different environments. Vertex genotypes, which are more responsive to environmental change, are specifically adapted genotypes that perform exceptionally well or poorly in some or all tested environments [7]. Based on the "which-won-where" biplot, the testing environments were divided into two MGEs with various high-performing genotypes for starch, protein, and Zn, but Fe only

had one MGE. Environments with the highest starch content for genotypes G4, G84, and G246 in MGE1 were MK20 and MK21, MS20 and MS21, and JM20, while MGE2 had environment JM21 with G265 as the highest starch content genotype. In terms of protein, MGE1 included environments MK21, MK20, MS20, JM21, and JM20 with genotype G161 having the highest protein content, whereas MGE2 only had environment MS21 with the genotype G358 having the highest protein content.

In terms of Zn, MGE1 included environments MK21, MK20, JM21, MS21, and JM20 with the genotype with the highest concentration of Zn, G264, whereas in MGE2 environment MS20 had genotypes with the highest concentration of Zn, G245, and G135. The highest Fe-containing genotypes, G137 and G142, were combined into a single MGE for all environments. This suggests that particular genotype adaptations to MGEs occurred, successfully exploiting the GE interaction [38]. The most effective way to take advantage of the positive GE interaction is to cluster the target environments into expressive MGEs and choose various genotypes for each MGE [15].

~~GGE biplots are ideal for graphically assessing testing environments, as they depict the heritability of traits based on vector length and genetic correlation between environments based on the angle between vectors—two critical components of test environment assessment [14]. This model is termed GGE because it accounts for both genotype effects and genotype-environment interaction~~ [12].

The most stable and high-ranking genotypes can be found using the GGE ranking biplot through AEC [12]. The highest starch content genotypes in the study were G246, G84, G4, and G175, while landraces with low starch contents like G264, G362, G355, and G212 experienced decreased stability due to the GEI effect. Genotypes G218, G43, and G161 scored highest in terms of protein content in this study. However, the GEI effect decreased the stability of landraces with high protein content, such as G358, G88, and G184. Although genotype G264 had the highest Zn content, the GEI effect made it less stable. The highest Fe content was found in G137 and G142. The GGE biplot model is effective for identifying top-ranking, stable genotypes across environments and selecting the best genotypes for specific mega-environments [39]. The current study found that the GGE biplot could distinguish between environments. The environments showed a similar level of discriminative power, as they were far from the biplot origin in both analyses. However, the contribution of the environments to genotype stability varied slightly.

Conclusion

The GGE biplot analysis allows for the examination of the relative stability and performance of genotypes across environments. This analysis identified specific genotypes with both high and stable starch, protein, Fe, and Zn content. Such genotypes are valuable for breeding programs aiming to develop varieties with consistent nutritional quality across diverse conditions.

These findings have practical implications for sorghum breeding programs, which address the nutritional needs of populations across diverse environments. To assess the adaptability of sorghum genotypes across diverse agroecological conditions, genotypes identified with both high and stable starch, protein, Fe, and Zn content, including G246, G84, G4, G175, G218, G43, G161, G137, G142 and G264, are deemed promising candidates for further evaluation and integration into breeding programs, particularly for their potential to provide nutritional stability across diverse agroecological zones. In this study, JM21 was the most discriminating test environment for starch content, while MS21 was the most discriminating for protein content.

The recommendations from this study emphasize a multifaceted approach to enhancing the adaptability and resilience of sorghum crops in diverse environments. This includes further evaluation through targeted breeding efforts and the consideration of agroecological factors.

Hence, the variability attributed to genotype and environment enables the selection of the most desirable traits in a specific environment. It is advisable to replicate this study across multiple environments to confirm and validate the obtained results.

Acknowledgments

We are grateful for the sorghum research staff at Melkassa Agricultural Research Center.

Author contribution

Chalachew Endalamaw: Conceptualization; data curation; investigation; methodology; visualization; formal analysis; writing – original draft; writing – review and editing. **Maryke Labuschagne**: Conceptualization; data curation; supervision; visualization; writing – review and editing. **Liezel Herselman**: Supervision; data curation; visualization; writing – review and editing.

Angeline van Biljon: Supervision; data curation; visualization; writing – review and editing. **Dagmawit Tsegaye**: Data curation; visualization; writing – review and editing.

Conflict of interest statement

The authors declare no conflict of interest.

ORCID

Chalachew Endalamaw https://orcid.org/0000-0002-5109-724X

Reference

1. Srinivasa Rao P, Reddy BV, Nagaraj N, Upadhyaya HD. Sorghum production for diversified uses. In: Wang Y-H, Upadhyaya HD, Kole C, editors. Genetics, genomics, and breeding of sorghum. 1st ed. CRC Press; 2014. https://doi.org/10.1201/b17153

2. Food and Agriculture Organization of the United Nations (FAO). FAOSTAT. Rome. http://faostat.fao.org . Accessed 01/12/2024.

3. Kaufman RC, Wilson JD, Bean SR, Galant AL, Perumal RR, Tesso T, Shi YC. Influence of genotype × location interaction on grain sorghum grain chemistry and digestibility. Agron J. 2018;110(5):1681-1688. https://doi.org/10.2134/agronj2017.09.0561

4. Ohanenye IC, Emenike CU, Mensi A, Medina-Godoy S, Jin J, Ahmed T, Sun X, Udenigwe CC. Food fortification technologies: Influence on iron, zinc, and vitamin A bioavailability and potential implications on micronutrient deficiency in sub-Saharan Africa. Sci Afr. 2021;11. https://doi.org/10.1016/j.sciaf.2020.e00667

5. Thakur NR, Gorthy S, Vemula A, Odeny DA, Ruperao P, Sargar PR, Mehtre SP, Kalpande HV, Habyarimana E. Genome-wide association study and expression of candidate genes for Fe and Zn concentration in sorghum grains. Sci Rep. 2024;14(1):12729. https://doi.org/10.1038/s41598-024-63308-0

6. Yan W, Tinker NA. Biplot analysis of multi-environment trial data: Principles and applications. Can J Plant Sci. 2006; 86:623-645. https://doi.org/10.4141/P05-169

7. Yan W, Kang MS, Ma B, Woods S, Cornelius PL. GGE biplot vs. AMMI analysis of genotype-by-environment data. Crop Sci. 2007; 47:643-653. https://doi.org/10.2135/cropsci2006.06.0374

8. Olanrewaju OS, Oyatomi O, Babalola OO, Abberton M. GGE biplot analysis of genotype × environment interaction and yield stability in Bambara groundnut. Agronomy. 2021;11(9):1839. https://doi.org/10.3390/agronomy11091839

9. Ninou E, Tsivelika N, Sistanis I, Katsenios N, Korpetis E, Vazaneli E, Papathanasiou F, Didos S, Argiriou A, Mylonas I. Assessment of Durum Wheat Cultivars' Adaptability to Mediterranean Environments Using G × E Interaction Analysis. Agronomy. 2023;14(1):102. https://doi.org/10.3390/agronomy14010102

10. Hemayati SS, Hamdi F, Saremirad A, Hamze H. Genotype by environment interaction and stability analysis for harvest date in sugar beet cultivars. Sci Rep. 2024;14(1):16015. https://doi.org/10.1038/s41598-024-67272-7

11. Finlay KW, Wilkinson GN. The analysis of adaptation in a plant-breeding program. Aust J Agric Res. 1963;14(6):742-754. https://doi.org/10.1071/AR9630742

12. Yan W. GGE biplot - A Windows application for graphical analysis of multi-environment trial data and other types of two-way data. Agron J. 2001;93:1111-1118. https://doi.org/10.2134/agronj2001.9351111x

13. Kang MS. Quantitative genetics, genomics, and plant breeding. Wallingford, UK: CAB International; 2003. https://doi.org/10.1079/9780851996011.0000

14. Yan W, Holland JB. A heritability-adjusted GGE biplot for test environment evaluation. Euphytica. 2010;171(3):355-369. https://doi.org/10.1007/s10681-009-0030-5

15. Yan W, Tinker NA. An integrated biplot analysis system for displaying, interpreting, and exploring genotype × environment interaction. Crop Sci. 2005;45:1004-1016. https://doi.org/10.2135/cropsci2004.0076

16. Yan W, Tinker NA. Biplot analysis of multi-environment trial data: Principles and applications. Can J Plant Sci. 2006;86:623-645. https://doi.org/10.4141/P05-169

17. Rao PS, Reddy PS, Rathore A, Reddy BV, Panwar S. Application of GGE biplot and AMMI model to evaluate sweet sorghum (Sorghum bicolor) hybrids for genotype × environment interaction and seasonal adaptation. Indian J Agric Sci. 2011;81:438-444.

18. Admas S, Tesfaye K. Genotype-by-environment interaction and yield stability analysis in sorghum (Sorghum bicolor (L.) Moench) genotypes in North Shewa, Ethiopia. Acta Univ Sapientiae Agric Environ. 2017;9:82-94. https://doi.org/10.1515/ausae-2017-0008

19. Worede F, Mamo M, Assefa S, Gebremariam T, Beze Y. Yield stability and adaptability of lowland sorghum (Sorghum bicolor (L.) Moench) in moisture-deficit areas of Northeast Ethiopia. Cogent Food Agric. 2020;6:1736865. https://doi.org/10.1080/23311932.2020.1736865

20. MoA (Ministry of Agriculture). Agro-ecological zones of Ethiopia: Natural resource management and regulatory department. Ethiopian Ministry of Agriculture, Addis Ababa, 1998. 1-28.

21. Redlands CA. ArcGIS Desktop: Release 10. Redlands: ESRI; 2011.

22. Cullis BR, Smith AB, Coombes NE. On the design of early generation variety trials with correlated data. J Agric Biol Environ Stat. 2006;11:381-393. https://doi.org/10.1198/108571106X154443

23. Butler DG, Cullis BR, Gilmour AR, Gogel BJ, Thompson R. ASReml-R reference manual version 4. VSN International Ltd, Hemel Hempstead, UK, 2017. pp. 30-49.

24. Smith AB, Borg LM, Gogel BJ, Cullis BR. Estimation of factor analytic mixed models for the analysis of multi-treatment multi-environment trial data. J Agric Biol Environ Stat. 2019;24:573-588. https://doi.org/10.1007/s13253-019-00362-6

25. Smith AB, Cullis BR. Plant breeding selection tools built on factor analytic mixed models for multi-environment trial data. Euphytica. 2018;214:1-19. https://doi.org/10.1007/s10681-018-2220-5

26. Horn L, Shimelis H, Sarsu F, Mwadzingeni L, Laing MD. Genotype-by-environment interaction for grain yield among novel cowpea (Vigna unguiculata L.) selections derived by gamma irradiation. Crop J. 2018;6:306-313. https://doi.org/10.1016/j.cj.2017.10.002

27. Zerihun M, Fox G, Nega A, Seyoum A, Minuye M, Jordan D, Taddese T, Assefa A. Near-Infrared Reflectance Spectroscopy (NIRS) for tannin, starch, and amylase determination in sorghum breeding programs. Int J Food Nutr Sci. 2020;7:45-50.

28. Singh S, Singh G, Singh P, Singh N. Effect of water stress at different stages of grain development on the characteristics of starch and protein of different wheat varieties. Food Chem. 2008;108:130-139. https://doi.org/10.1016/j.foodchem.2007.10.054

29. Gunaratne A, Ratnayaka UK, Sirisena N, Ratnayaka J, Kong XL, Arachchi LV, Corke H. Effect of soil moisture stress from flowering to grain maturity on functional properties of Sri Lankan rice flour. Starke. 2011;63:283-290. https://doi.org/10.1002/star.201000108

30. Fredriksson H, Silverio J, Andersson R, Eliasson AC, Aman P. The influence of amylose and amylopectin characteristics on gelatinization and retrogradation properties of different starches. Carbohydr Polym. 1998;35:119-134. https://doi.org/10.1016/S0144-8617(97)00247-6

31. Cepeda M, Waniska RD, Rooney LW, Bejosano FP. Effects of leavening acids and dough temperature in wheat flour tortillas. Cereal Chem. 2000;77(4):489-494. https://doi.org/10.1094/CCHEM.2000.77.4.489

32. Phuke RM, Anuradha K, Radhika K, Jabeen F, Anuradha G, Ramesh T, Hariprasanna K, Mehtre SP, Deshpande SP, Anil G. Genetic variability, genotype × environment interaction, correlation and GGE biplot analysis for grain iron and zinc concentration and other agronomic traits in RIL population of sorghum (Sorghum bicolor L. Moench). Front Plant Sci. 2017;8:712. https://doi.org/10.3389/fpls.2017.00712

33. Gregorio GB. Progress in breeding for trace minerals in staple crops. J Nutr. 2002;132:500S-502S. https://doi.org/10.1093/jn/132.3.500S

34. Stangoulis JC, Knez M. Biofortification of major crop plants with iron and zinc - Achievements and future directions. Plant Soil. 2022;474:57-76. https://doi.org/10.1007/s11104-022-05330-7

35. Boyles RE, Cooper EA, Myers MT, Brenton Z, Rauh BL, Morris GP, Kresovich S. Genome-wide association studies of grain yield components in diverse sorghum germplasm. Plant Genome. 2016;9:1-17. https://doi.org/10.3835/plantgenome2015.09.0091

36. Rakshit S, Ganapathy KN, Gomashe SS, Rathore A, Ghorade RB, Kumar MN, Patil JV. GGE biplot analysis to evaluate genotype, environment, and their interactions in sorghum multi-location data. Euphytica. 2012;185:465-479. https://doi.org/10.1007/s10681-012-0648-6

37. Enyew M, Feyissa T, Geleta M, Tesfaye K, Hammenhag C, Carlsson AS. Genotype by environment interaction, correlation, AMMI, GGE biplot, and cluster analysis for grain yield and other agronomic traits in sorghum (Sorghum bicolor L. Moench). PLoS One. 2021;16(10). https://doi.org/10.1371/journal.pone.0258211

38. Mohammadi M, Noorinia AA, Khalilzadeh GR, Hosseinpoor T. Application of GGE biplot analysis to investigate GE interaction on barley grain yield. Curr Opin Agric. 2015;4:25.

39. Yan W, Kang MS. GGE biplot analysis: A graphical tool for breeders, geneticists, and agronomists. CRC Press; 2002. pp. 63-83. https://doi.org/10.1201/9781420040371

Appendices

Appendix 1. Data on monthly temperatures and rainfall for two consecutive seasons (2020 and 2021).

Year	Month	Melkassa				Jimma				Miesso			
		Rainfall (mm)	Temperature °C			Rainfall (mm)	Temperature °C			Rainfall (mm)	Temperature °C		
			Minimum	Maximum	Mean		Minimum	Maximum	Mean		Minimum	Maximum	Mean
2020	January	0.20	12.40	27.10	19.70	28.70	9.50	27.30	18.40	0.00	13.80	28.00	20.90
2020	February	0.00	15.00	30.20	22.60	38.40	9.50	28.90	19.20	1.20	14.80	30.40	22.60
2020	March	83.00	16.10	32.20	24.20	35.00	8.90	29.30	19.10	5.70	17.40	32.50	24.95
2020	April	84.40	16.30	31.00	23.70	33.70	10.60	28.00	19.30	176.60	18.50	30.60	24.55
2020	May	45.90	16.40	30.80	23.60	105.60	9.70	26.90	18.30	83.10	18.40	32.00	25.20
2020	June	183.50	17.40	29.90	23.70	154.20	10.30	28.00	19.10	44.30	18.90	33.70	26.30
2020	July	327.70	15.60	24.70	20.10	119.40	10.00	26.90	18.50	225.50	18.00	29.70	23.85
2020	August	304.50	15.00	23.70	19.40	150.90	10.40	26.50	18.50	198.60	18.80	29.30	24.05
2020	September	54.40	16.00	27.60	21.80	162.60	9.90	25.90	17.90	69.50	16.50	29.90	23.20
2020	October	1.50	10.70	29.70	20.20	210.20	10.10	27.30	18.70	6.00	12.70	31.80	22.25
2020	November	0.00	10.90	28.80	19.90	217.30	10.30	28.70	19.50	14.50	12.20	30.10	21.15
2020	December	0.00	9.40	28.20	18.80	273.80	9.80	27.50	18.70	0.70	10.80	29.00	19.90
2021	January	0.00	9.90	28.40	19.20	349.20	9.80	26.80	18.30	0.00	9.70	27.90	18.80
2021	February	33.70	12.30	28.50	20.40	115.40	10.60	27.50	19.10	0.00	12.80	29.60	21.20
2021	March	0.00	15.50	32.60	24.00	38.70	10.40	28.00	19.20	9.80	14.30	32.70	23.50
2021	April	76.20	15.70	32.40	24.10	3.30	10.70	28.30	19.50	101.80	17.20	32.60	24.90
2021	May	28.30	16.70	31.40	24.10	173.50	11.00	27.80	19.40	135.90	17.40	31.20	24.30
2021	June	40.20	17.10	32.50	24.80	179.00	12.10	28.30	20.20	0.00	18.70	34.00	26.35
2021	July	91.50	16.40	26.00	21.20	300.20	11.90	27.50	19.70	163.80	18.40	28.80	23.60
2021	August	238.60	15.90	27.80	21.90	182.20	11.80	26.70	19.30	95.60	17.80	29.90	23.85
2021	September	69.40	15.40	27.60	21.50	316.00	11.50	26.00	18.70	69.90	17.00	29.50	23.25
2021	October	51.70	12.00	28.60	20.30	169.10	11.10	26.10	18.60	51.90	13.80	30.60	22.20
2021	November	12.40	10.60	29.40	20.00	266.80	10.40	27.40	18.90	0.00	10.70	30.80	20.75
2021	December	0.00	10.00	28.60	19.30	166.80	10.70	28.30	19.50	-	-	-	-

Appendix 2. Mean (BLUP) quality traits performance of the 365 sorghum landraces across four environments.

Code	Genotypes	Protein	Starch	AM	AMP	Fe	Zn	Ash	MST
G1	Adukar	8.57	68.36	19.1	80.9	35.76	37.09	2.91	15.57
G2	Assosa-1	8.69	68.25	19.16	80.84	33.65	33.2	2.27	15.58
G3	B35	8.18	69.41	19.68	80.32	24.2	26.45	1.58	15.26
G4	Bonsa	8.4	69.65	19.84	80.16	25.69	23.33	1.29	15.36
G5	Dagim	9.07	69.09	19.76	80.24	20.74	24.14	1.31	15.45
G6	DS10	9.67	69.11	19.71	80.29	12.08	24.87	1.48	15.5
G7	ETSL100005	9.64	68.86	19.55	80.46	49.17	29.14	1.88	15.48
G8	ETSL100007	8.26	68.77	19.54	80.46	38.57	33.75	2.3	15.61
G9	ETSL100016	9.05	68.83	19.74	80.26	53.29	32.09	2.05	15.48
G10	ETSL100020	9.17	68.59	19.56	80.45	38.25	28.8	1.89	15.74
G11	ETSL100022	9.56	68.74	19.6	80.4	32.93	28.06	1.81	15.5
G12	ETSL100023	10.27	68.36	19.14	80.86	30.58	28.67	2.08	15.77
G13	ETSL100025	8.17	68.91	19.43	80.57	39.92	32.65	2.16	15.63
G14	ETSL100030	9.44	69	19.76	80.24	31.31	24.87	1.45	15.4
G15	ETSL100034	8.75	69.11	19.47	80.53	28.94	27.27	1.67	15.63
G16	ETSL100039	9.18	67.96	19.71	80.29	51.48	34.23	2.11	15.63
G17	ETSL100040	9.17	68.86	19.49	80.52	46.91	29.92	1.93	15.48
G18	ETSL100044	9.66	68.79	19.75	80.25	13.05	23.78	1.55	15.33
G19	ETSL100046	9.09	68.38	19.34	80.67	30.82	31.31	2	15.71
G20	ETSL100048	9.41	68.49	19.46	80.54	25.31	26.91	1.69	15.72
G21	ETSL100060	9.78	68.8	19.67	80.33	40.23	25.72	1.58	15.53
G22	ETSL100062	9.52	68.67	19.28	80.73	39.97	27.46	1.96	15.95
G23	ETSL100063	8.3	68.81	19.42	80.58	57.84	31.96	2.45	15.49
G24	ETSL100064	8.83	68.78	19.45	80.55	33.8	29.96	1.86	15.43
G25	ETSL100066	8.15	68.32	19.22	80.78	35.15	32.05	2.2	15.85
G26	ETSL100068	8.76	68.28	19.45	80.55	40.26	34.44	2.29	15.72
G27	ETSL100072	9.35	68.59	19.39	80.61	27.64	27.49	2.01	15.94
G28	ETSL100078	9.5	68.91	19.58	80.42	57.02	29.71	2.01	15.37
G29	ETSL100079	8.72	68.68	19.46	80.55	37.44	29.85	1.94	15.77
G30	ETSL100090	8.67	68.26	19.43	80.57	39.55	29.61	1.91	16.03
G31	ETSL100091	8.89	68.6	19.45	80.55	21.38	28.79	2.3	15.82
G32	ETSL100093	10.29	68.49	19.73	80.28	34.34	25.02	1.52	15.47
G33	ETSL100096	9.28	68.96	19.78	80.22	19.43	25.4	1.73	15.52
G34	ETSL100100	8.62	68.86	19.49	80.51	19.3	28.72	2.08	15.99

G35	ETSL100105	9.78	68.7	19.28	80.72	38.92	30.09	2.13	15.58
G36	ETSL100107	8.95	68.82	19.44	80.56	40.01	28.62	1.96	15.5
G37	ETSL100112	9.16	68.97	19.45	80.55	41.05	28.99	1.92	15.45
G38	ETSL100120	10.28	68.63	19.39	80.61	29.3	27.23	1.82	15.71
G39	ETSL100121	10.07	68.29	19.37	80.63	35.75	28.97	1.72	15.51
G40	ETSL100127	9.25	69.22	19.49	80.51	30.86	27.67	1.74	15.65
G41	ETSL100141	8.9	69.33	19.64	80.36	46.91	30.87	1.91	15.52
G42	ETSL100149	9.56	68.74	19.6	80.4	32.93	28.06	1.81	15.5
G43	ETSL100150	10.35	68.53	19.62	80.39	16.97	25.79	1.79	15.52
G44	ETSL100152	10.41	68.76	19.5	80.51	26.72	23.96	1.55	15.64
G45	ETSL100167	9.65	68.6	19.62	80.38	48.09	29.61	2.01	15.46
G46	ETSL100169	9.5	68.37	19.09	80.91	33.77	33.22	2.38	15.64
G47	ETSL100170	8.54	69.2	19.6	80.41	33.2	27.52	1.82	15.55
G48	ETSL100178	9.68	67.93	19.04	80.97	34.54	31.46	2.41	15.91
G49	ETSL100181	9.91	68.47	19.29	80.71	34.72	29.5	1.97	15.95
G50	ETSL100184	9.91	68.52	19.59	80.41	19.13	25.88	1.71	15.64
G51	ETSL100192	9.44	68.39	19.45	80.55	24.75	28.49	1.82	15.63
G52	ETSL100198	9.85	68.36	19.35	80.66	34.79	27.32	1.78	15.79
G53	ETSL100200	9.23	68.84	19.67	80.33	33.81	27.87	1.59	15.78
G54	ETSL100204	10.36	68.46	19.79	80.21	26.84	25.6	1.38	15.59
G55	ETSL100214	9.25	69.24	19.66	80.34	59.69	28.8	2.13	15.31
G56	ETSL100233	9.4	68.96	19.82	80.18	35.44	27.47	1.66	14.89
G57	ETSL100234	9.43	68.47	19.59	80.41	47.74	30.85	2.04	15.83
G58	ETSL100238	10.05	68.23	19.25	80.75	25.7	28.07	1.89	15.81
G59	ETSL100246	9.16	68.93	19.59	80.41	42.83	24.77	1.75	15.57
G60	ETSL100249	10.21	68.29	19.38	80.62	30.98	27.35	1.6	15.52
G61	ETSL100250	9.18	68.96	19.52	80.48	49.84	30.65	2.11	15.17
G62	ETSL100267	9.94	68.13	19.27	80.73	25.07	29.62	1.71	15.74
G63	ETSL100275	8.09	69.47	19.61	80.39	40.77	29.64	1.7	15.5
G64	ETSL100284	9.27	68.79	19.53	80.48	15.55	24.93	1.49	15.47
G65	ETSL100286	9.25	69.01	19.5	80.5	43.11	26.84	1.91	15.52
G66	ETSL100292	9.52	68.8	19.57	80.43	24.08	26.79	1.87	15.59
G67	ETSL100297	10.07	68.68	19.67	80.34	28.47	25.54	1.5	15.56
G68	ETSL100303	9.89	69.04	19.7	80.3	36.32	26.28	1.59	15.54
G69	ETSL100307	9.18	69.16	19.55	80.45	20.55	31.86	1.91	15.74
G70	ETSL100310	9.69	68.92	19.57	80.43	37.08	25.32	1.63	15.52
G71	ETSL100315	9.33	69.06	19.53	80.47	27.69	27.62	1.93	15.43

G72	ETSL100328	8.85	69.15	19.74	80.26	43.22	28.86	1.72	15.32
G73	ETSL100332	9.29	68.43	19.27	80.73	36.39	30.93	2.18	15.66
G74	ETSL100340	10.02	68.76	19.55	80.45	39.72	26.67	1.93	15.53
G75	ETSL100349	10.08	68.18	19.61	80.39	28.67	26.26	1.33	15.71
G76	ETSL100350	10.17	68.11	19.76	80.25	23.61	26.55	1.46	15.46
G77	ETSL100353	8.33	69.09	19.6	80.4	28.09	30.47	1.85	15.25
G78	ETSL100365	10.2	68.77	19.55	80.45	23.52	23.77	1.28	15.64
G79	ETSL100366	9.56	68.74	19.6	80.4	32.93	28.06	1.81	15.5
G80	ETSL100367	9.44	68.95	19.5	80.5	21.46	26.29	1.81	15.6
G81	ETSL100388	9.19	68.49	19.4	80.61	36.6	29.12	1.97	15.5
G82	ETSL100392	10.06	68.54	19.51	80.49	19.69	27.56	1.8	15.5
G83	ETSL100400	9.33	68.97	19.6	80.4	41.13	28.79	1.86	15.22
G84	ETSL100406	9.44	69.56	19.83	80.18	30.63	25.64	1.53	15.21
G85	ETSL100408	10.17	68.45	19.68	80.33	27.41	34.98	2.07	15.72
G86	ETSL100409	10	68.57	19.52	80.48	22.86	27.15	1.89	15.52
G87	ETSL100417	9.45	69.26	19.6	80.4	57.79	32.84	2.31	15.3
G88	ETSL100419	10.75	68.44	19.38	80.62	32.04	24.56	1.85	15.69
G89	ETSL100427	10.09	68.96	19.66	80.34	20.69	25.86	1.71	15.47
G90	ETSL100428	9.27	67.88	19.61	80.39	29.97	26.73	1.66	15.97
G91	ETSL100430	10.55	68.87	19.47	80.53	26.42	24.41	1.59	15.83
G92	ETSL100431	9.82	68.6	19.73	80.27	19.98	26.03	1.66	15.58
G93	ETSL100432	9.36	69.1	19.72	80.29	21.86	24.54	1.53	15.43
G94	ETSL100436	7.99	68.74	19.5	80.5	43.66	32.97	2.18	15.5
G95	ETSL100438	9.57	68.62	19.67	80.33	40.82	28.51	1.58	15.49
G96	ETSL100443	9.33	69.11	19.84	80.16	23.76	23.7	1.22	15.58
G97	ETSL100444	9.73	68.87	19.82	80.18	30.9	24.87	1.39	15.32
G98	ETSL100445	8.88	68.69	19.41	80.6	39.18	32.77	2.39	15.6
G99	ETSL100450	8.96	68.88	19.67	80.32	52.52	29.15	2.03	15.44
G100	ETSL100465	8.84	68.68	19.51	80.49	29.95	28.64	2.11	15.72
G101	ETSL100474	9.11	69.09	19.5	80.5	23.81	24.94	1.71	15.77
G102	ETSL100476	9.63	68.79	19.73	80.27	18.97	23.97	1.39	15.63
G103	ETSL100485	9.57	68.89	19.75	80.25	34.34	24.74	1.5	15.64
G104	ETSL100488	9.77	68.88	19.74	80.27	35.76	25.39	1.55	15.2
G105	ETSL100491	9.12	68.42	19.54	80.46	25.22	28.95	1.88	15.81
G106	ETSL100492	8.59	68.8	19.88	80.12	35.43	27.94	1.58	15.93
G107	ETSL100494	10.07	68.55	19.68	80.32	34.68	25.52	1.57	15.5
G108	ETSL100502	9.75	68.95	19.81	80.2	32.33	24.45	1.54	15.21

G109	ETSL100529	9.87	68.36	19.53	80.47	22.43	28.77	1.81	15.27
G110	ETSL100539	9.35	68.4	19.6	80.4	28.55	29.86	1.8	15.31
G111	ETSL100544	10.28	68.57	19.59	80.41	20.95	22.99	1.34	15.59
G112	ETSL100550	10.06	68.95	19.63	80.37	19.19	26.51	1.91	15.42
G113	ETSL100560	10.25	68.62	19.73	80.27	32.37	25.82	1.44	15.7
G114	ETSL100561	9.61	68.88	19.69	80.31	35.9	25.11	1.65	15.51
G115	ETSL100565	8.7	68.97	19.74	80.26	45.42	26.49	1.75	15.44
G116	ETSL100568	8.4	68.86	19.85	80.15	45.53	34.74	2.07	15.35
G117	ETSL100575	9.01	69.06	19.85	80.15	47.98	30.6	2.04	15.31
G118	ETSL100584	9.54	68.61	19.27	80.73	29.93	27.56	1.83	15.53
G119	ETSL100587	8.53	68.91	19.71	80.3	49.33	32.25	2.03	14.9
G120	ETSL100593	9.64	69.13	19.68	80.32	38.1	26.16	1.81	15.28
G121	ETSL100619	9.97	68.92	19.69	80.31	14.9	25.51	1.53	15.58
G122	ETSL100620	9.24	69.31	19.98	80.02	22.93	24.11	1.28	15.31
G123	ETSL100634	9.01	69.41	19.79	80.21	45.2	26.43	1.79	15.14
G124	ETSL100638	9.19	69.11	19.63	80.37	37.82	25.96	1.76	15.46
G125	ETSL100639	9.65	68.81	19.47	80.53	20.39	25.84	1.55	15.94
G126	ETSL100642	9.45	68.49	19.32	80.68	26.3	29.62	1.76	15.78
G127	ETSL100644	10.05	68.75	19.8	80.21	22.12	25.84	1.39	15.48
G128	ETSL100648	10.55	68.85	19.61	80.4	16.77	24.62	1.57	15.8
G129	ETSL100651	9.56	68.74	19.6	80.4	32.93	28.06	1.81	15.5
G130	ETSL100661	10.24	68.6	19.75	80.25	23.69	24.77	1.45	15.57
G131	ETSL100666	9.64	69.05	19.65	80.35	35.39	26.53	1.81	15.49
G132	ETSL100667	10.38	68.65	19.64	80.36	15.24	25.7	1.64	15.32
G133	ETSL100669	9.29	68.4	19.29	80.71	36.29	32.09	2.13	15.48
G134	ETSL100683	9.12	68.93	19.82	80.18	23.59	25.55	1.44	15.48
G135	ETSL100689	9.38	68.98	19.84	80.16	49.07	31.36	1.99	14.6
G136	ETSL100699	9.49	68.98	19.73	80.27	29.4	23.98	1.39	15.59
G137	ETSL100702	9.82	69.3	19.88	80.12	79.56	38.1	2.61	15.41
G138	ETSL100707	9.9	68.86	19.78	80.22	38.82	24.88	1.66	15.6
G139	ETSL100716	9.27	68.95	19.78	80.22	23.14	23.65	1.23	15.22
G140	ETSL100720	9.41	68.84	19.66	80.34	52.69	34.46	2.2	15.22
G141	ETSL100722	10.52	68.52	19.65	80.36	40.95	24.52	1.47	15.52
G142	ETSL100735	7.97	69.13	20.47	79.53	80.77	50.03	2.95	15.73
G143	ETSL100739	10.77	68.25	19.52	80.48	39.9	27.13	1.68	15.55
G144	ETSL100745	9.67	69	19.6	80.4	48.49	31.35	2.09	15.37
G145	ETSL100751	10.12	68.96	19.63	80.37	38.08	25.38	1.79	15.24

G146	ETSL100761	10.26	68.42	19.54	80.45	49.66	33.99	2.19	15.43
G147	ETSL100763	9.59	68.54	19.57	80.43	38.71	29.81	1.77	15.27
G148	ETSL100768	9.2	68.41	20.02	79.98	53.51	44.31	2.96	15.3
G149	ETSL100771	10.13	68.88	19.73	80.27	57.45	34.07	2.21	15.08
G150	ETSL100782	10.13	68.27	19.66	80.34	32.26	24.49	1.56	15.45
G151	ETSL100785	10.3	69	19.5	80.5	31.5	25.2	1.79	15.67
G152	ETSL100786	9.26	69.14	19.76	80.24	36.06	24.34	1.65	15.3
G153	ETSL100792	10.3	68.39	19.5	80.5	24.57	25.97	1.9	15.03
G154	ETSL100797	10.12	68.59	19.65	80.35	41.2	26.48	1.65	15.5
G155	ETSL100798	9.1	68.82	19.61	80.39	33.5	26.08	1.57	15.43
G156	ETSL100801	9.55	69.05	19.73	80.27	18.53	25.35	1.76	15.47
G157	ETSL100805	10.1	68.69	19.56	80.44	35.51	25.24	1.88	15.27
G158	ETSL100808	9.5	68.98	19.72	80.28	39.17	24.47	1.73	15.22
G159	ETSL100813	10.51	68.61	19.56	80.44	29.97	24.76	1.65	15.4
G160	ETSL100825	9	69.31	19.74	80.27	33.97	24.56	1.42	15.38
G161	ETSL100834	10.99	68.15	19.63	80.37	34.58	26.37	1.53	15.49
G162	ETSL100837	9.91	69.38	19.65	80.35	33.28	25.33	1.65	15.08
G163	ETSL100841	8.8	68.38	19.96	80.04	29.39	34.12	1.78	14.46
G164	ETSL100842	9	68.15	19.59	80.41	47.24	33.9	1.96	15.66
G165	ETSL100851	9.07	68.89	19.73	80.27	38.38	26.93	1.61	15.55
G166	ETSL100861	9.84	69.01	19.73	80.27	63.09	34.66	2.45	15.13
G167	ETSL100862	9.25	69.25	19.8	80.2	27.36	24.56	1.43	15.33
G168	ETSL100863	8.78	68.93	19.68	80.32	40.98	28.96	1.88	15.13
G169	ETSL100867	9.03	68.32	19.67	80.33	29.61	29.17	1.51	15.61
G170	ETSL100872	9.23	68.77	19.64	80.36	38.12	29.74	1.78	15.26
G171	ETSL100874	10.95	68.85	19.65	80.35	24.73	25.16	1.57	15.36
G172	ETSL100876	9.49	69.12	19.87	80.13	59.99	27.76	1.97	15.38
G173	ETSL100877	9.84	67.98	19.42	80.58	30.19	31.16	2.14	15.95
G174	ETSL100888	9.74	68.19	19.76	80.24	45.16	30.43	1.94	15.57
G175	ETSL100889	8.61	69.55	20.3	79.7	52.71	46.47	2.51	15.04
G176	ETSL100890	10.3	68.51	19.6	80.4	34.11	27.58	1.65	15.62
G177	ETSL100893	9.42	68.91	19.67	80.33	20.19	25.39	1.71	15.39
G178	ETSL100895	8.68	69.2	19.48	80.52	38.66	29.04	1.95	15.47
G179	ETSL100898	9.1	69.08	19.78	80.22	44.66	26.46	1.63	15.41
G180	ETSL100900	10.16	68.63	19.53	80.47	51.72	32.75	2.19	15.43
G181	ETSL100902	9.97	68.73	19.63	80.37	30.78	31.16	2.17	15.35
G182	ETSL100916	8.88	68.71	19.52	80.48	50.6	30.73	2.04	15.6

G183	ETSL100919	9.14	68.66	19.29	80.71	30.97	26.86	1.79	15.9
G184	ETSL100921	10.84	68.58	19.21	80.79	20.92	27.03	1.84	16.08
G185	ETSL100928	10.11	68.65	19.54	80.46	27.16	23.7	1.5	15.5
G186	ETSL100945	9.7	69.19	19.55	80.45	25.57	23.49	1.52	15.68
G187	ETSL100951	9.06	68.78	19.5	80.5	30.19	27.39	1.87	15.62
G188	ETSL100953	10.24	68.45	19.51	80.5	22.62	26.16	1.92	15.62
G189	ETSL100956	9.6	68.85	19.58	80.42	24.65	24.65	1.72	15.75
G190	ETSL100964	9.74	68.5	19.44	80.56	29.3	30.92	2.02	15.37
G191	ETSL100970	9.56	68.74	19.6	80.4	32.93	28.06	1.81	15.5
G192	ETSL100977	10.04	68.63	19.41	80.59	28.18	29.94	2.01	15.82
G193	ETSL100978	8.61	68.83	19.74	80.26	52.28	32.59	1.82	15.44
G194	ETSL100983	8.72	69.44	19.68	80.32	47.88	29.88	2.02	15.22
G195	ETSL100985	10.2	68.29	19.59	80.41	34.23	27.53	1.64	15.18
G196	ETSL101000	10.26	68.36	19.48	80.53	27.28	26.37	1.64	15.51
G197	ETSL101001	9.84	68.65	19.74	80.26	18.85	26.89	1.76	15.61
G198	ETSL101006	9.12	68.93	19.69	80.31	16.25	26.1	1.74	15.75
G199	ETSL101014	9.66	68.57	19.42	80.58	20.3	30.04	1.81	15.38
G200	ETSL101015	9.64	68.62	19.59	80.41	19.58	27.48	1.89	15.55
G201	ETSL101019	9.37	68.79	19.69	80.31	23.35	25.65	1.6	15.3
G202	ETSL101021	7.69	69.19	19.8	80.2	23.96	30.88	2.06	15.41
G203	ETSL101023	9.94	68.97	19.46	80.54	20.29	25.51	1.76	15.77
G204	ETSL101029	9.21	68.74	19.58	80.42	20.42	27.54	1.79	15.74
G205	ETSL101038	9.7	68.88	19.61	80.39	19.11	24.25	1.66	15.74
G206	ETSL101051	8.53	68.52	19.37	80.63	45.24	37.23	2.39	15.66
G207	ETSL101056	8.27	67.92	19.66	80.34	30.75	28.64	1.66	14.44
G208	ETSL101058	9.5	68.48	19.31	80.69	21.22	28.26	1.88	16.01
G209	ETSL101059	9.79	68.39	19.54	80.46	23.25	26.5	1.72	15.55
G210	ETSL101061	9.18	68.67	19.67	80.33	33.63	27.52	1.93	15.5
G211	ETSL101074	9.7	69.02	19.65	80.35	33.79	25.84	1.55	15.56
G212	ETSL101081	10.39	67.63	19.47	80.53	50.16	34.68	2.26	15.56
G213	ETSL101084	10.02	68.85	19.61	80.39	19.87	25.09	1.8	15.71
G214	ETSL101097	10.64	68.46	19.58	80.43	31.99	25.08	1.78	15.55
G215	ETSL101101	9.25	68.72	19.71	80.29	42.98	28.93	1.72	15.17
G216	ETSL101110	9.73	68.92	19.64	80.36	24.24	25.5	1.46	15.48
G217	ETSL101114	10.23	68.84	19.68	80.32	35.04	23.84	1.6	15.28
G218	ETSL101118	10.84	68.47	19.48	80.52	20.17	27.11	1.76	15.43
G219	ETSL101127	9.27	69.05	19.61	80.39	21.47	25.47	1.72	15.73

G220	ETSL101128	10.01	68.4	19.53	80.47	15.77	26.66	1.88	15.86
G221	ETSL101138	10.86	68.35	19.39	80.62	22.27	27.53	2.05	15.67
G222	ETSL101142	10.22	68.58	19.68	80.32	37.16	26.05	1.54	15.47
G223	ETSL101160	9.72	68.87	19.68	80.32	35.55	25.74	1.61	15.55
G224	ETSL101161	9.09	68.92	19.65	80.35	25.52	26.49	1.89	15.32
G225	ETSL101163	10.49	68.08	19.25	80.75	20.41	28.91	2.06	16.02
G226	ETSL101173	9.59	68.9	19.41	80.59	29.58	26.93	1.68	15.66
G227	ETSL101180	9.96	68.33	19.54	80.46	19.09	25.71	1.74	15.38
G228	ETSL101181	9.89	68.28	19.47	80.53	25.18	28.93	1.7	15.74
G229	ETSL101190	10.06	68.78	19.48	80.52	28.12	26.43	1.8	15.58
G230	ETSL101199	8.61	68.8	19.96	80.04	40.26	38.98	2.36	14.66
G231	ETSL101206	10.52	68.61	19.57	80.43	24.31	26.52	1.67	15.47
G232	ETSL101210	10.1	68.47	19.63	80.37	29.83	28.22	1.81	15.54
G233	ETSL101215	10.17	68.43	19.71	80.29	35.32	25.2	1.77	15.43
G234	ETSL101217	9.13	69.1	19.82	80.18	34.06	24.92	1.45	15.34
G235	ETSL101218	8.7	69.25	19.71	80.29	23.79	27.83	1.66	15.34
G236	ETSL101224	9.82	68.78	19.73	80.27	46.49	27.14	1.84	15.02
G237	ETSL101225	9.66	68.85	19.62	80.39	33.69	27.99	1.8	15.78
G238	ETSL101231	10.04	68.7	19.86	80.15	21.33	25.53	1.45	15.35
G239	ETSL101232	9.04	68.56	19.46	80.54	47.24	34.7	2.06	15.76
G240	ETSL101246	9.43	69.13	19.66	80.34	30.2	26.31	1.47	15.6
G241	ETSL101247	9.86	68.74	19.63	80.37	28.23	31.82	1.88	15.37
G242	ETSL101248	9.41	68.9	19.6	80.4	36.63	26.38	1.58	15.43
G243	ETSL101249	10.07	68.49	19.41	80.59	49.05	28.85	1.94	15.43
G244	ETSL101254	9.98	68.94	19.88	80.12	29.8	24.11	1.23	15.58
G245	ETSL101255	8.81	68.15	19.74	80.26	45.65	30.96	1.89	14.35
G246	ETSL101256	8.98	69.6	19.83	80.18	40.14	25.39	1.68	15.32
G247	ETSL101258	9.56	69.12	19.7	80.31	38.03	24.7	1.56	15.65
G248	ETSL101262	8.83	69.28	19.79	80.21	62.91	30.14	2.16	15.21
G249	ETSL101270	9.28	69.33	19.74	80.26	35.35	24.41	1.64	15.32
G250	ETSL101274	9.39	69.09	19.79	80.21	32.78	24.46	1.59	15.34
G251	ETSL101277	9.11	68.35	19.57	80.43	40.88	31.43	2.09	15.13
G252	ETSL101289	9.96	68.65	19.39	80.61	28.48	28.73	1.8	15.66
G253	ETSL101292	9.46	69.26	19.6	80.4	31.38	29.67	1.65	15.42
G254	ETSL101293	9.86	68.86	19.63	80.37	36.8	25.26	1.68	15.47
G255	ETSL101297	9.6	68.61	19.62	80.37	18.54	26.62	1.55	15.52
G256	ETSL101299	9.3	69.13	19.61	80.39	23.61	26.07	1.49	15.36

G257	ETSL101305	8.88	69.24	19.69	80.31	38.47	26.8	1.63	15.51
G258	ETSL101310	10.11	68.84	19.55	80.45	48.25	28.01	2.01	15.73
G259	ETSL101312	9.09	68.66	19.66	80.34	54.08	37.5	2.38	15.29
G260	ETSL101313	9.71	68.78	19.52	80.48	61.4	30.7	2.03	15.26
G261	ETSL101316	8.81	69.25	19.71	80.3	45.2	26.23	1.76	15.48
G262	ETSL101321	10.21	68.42	19.56	80.44	26.68	25.12	1.72	15.41
G263	ETSL101323	9.9	68.68	19.71	80.29	30.21	24.08	1.36	15.41
G264	ETSL101325	9.72	67.84	20.66	79.34	40.01	58.04	2.98	15.38
G265	ETSL101335	8.02	69.26	19.56	80.44	36.3	26.31	1.78	15.63
G266	ETSL101337	9.33	68.46	19.62	80.38	35.09	27.1	1.58	15.71
G267	ETSL101346	9.43	68.84	19.5	80.5	25.97	25.4	1.73	15.58
G268	ETSL101347	9.95	68.66	19.56	80.45	20.47	25.36	1.61	15.34
G269	ETSL101353	9.71	68.65	19.58	80.42	30.5	29.56	1.96	15.6
G270	ETSL101358	9.81	68.45	19.6	80.4	29.14	27.93	1.64	15.53
G271	ETSL101362	8.43	68.6	19.3	80.7	40.14	33.78	2.13	15.53
G272	ETSL101369	9.31	68.86	19.68	80.32	12.96	25.16	1.72	15.33
G273	ETSL101388	10.14	68.13	19.51	80.49	21.64	29.43	1.99	15.74
G274	ETSL101389	10.28	68.64	19.63	80.37	21.03	26.71	1.74	15.7
G275	ETSL101396	9.87	68.67	19.64	80.36	16.32	24.27	1.51	15.51
G276	ETSL101404	8.92	69.04	19.65	80.35	46.16	30.81	2	15.21
G277	ETSL101408	8.13	68.71	19.37	80.64	33.11	30.04	2	15.62
G278	ETSL101410	9.72	68.56	20.49	79.51	46.44	48.24	2.68	14.38
G279	ETSL101416	9.59	68.95	19.66	80.34	59.47	29.71	2.06	15.04
G280	ETSL101418	9.68	69.04	19.59	80.41	22.21	25.52	1.88	15.48
G281	ETSL101431	9.61	68.81	19.57	80.43	22.04	26.61	1.74	15.65
G282	ETSL101436	10.34	68.17	19.59	80.41	23.97	26.13	1.86	15.65
G283	ETSL101443	10.37	68.45	19.51	80.49	26.74	25.71	1.79	15.59
G284	ETSL101444	10.01	68.48	19.48	80.52	25.99	24.97	1.71	15.81
G285	ETSL101445	9.3	68.77	19.56	80.44	50.33	28.26	1.93	15.63
G286	ETSL101446	8.98	68.98	19.82	80.18	25.92	28.29	1.96	15.66
G287	ETSL101451	10.18	68.58	19.57	80.43	33.55	26.19	1.69	15.45
G288	ETSL101453	10.58	68.44	19.54	80.46	34.94	28.16	1.63	15.43
G289	ETSL101459	10	68.87	19.67	80.33	32.29	22.92	1.5	15.52
G290	ETSL101461	9.93	68.21	19.42	80.58	21.72	28.28	1.81	16.05
G291	ETSL101464	9.23	68.86	19.61	80.39	17.39	26.5	1.87	15.52
G292	ETSL101470	10.1	68.41	19.63	80.37	17.84	24.62	1.89	15.62
G293	ETSL101477	10.78	68.29	19.48	80.52	23.99	26.81	1.87	15.48

G294	ETSL101486	10.45	68.14	19.13	80.87	24.27	29.72	1.88	15.64
G295	ETSL101491	10.28	68.69	19.59	80.41	22.22	25.94	1.67	15.37
G296	ETSL101492	9.94	68.97	19.69	80.31	34.04	21.92	1.73	15.44
G297	ETSL101506	10.31	68.6	19.73	80.27	29.7	26.65	1.55	15.51
G298	ETSL101508	9.68	68.9	19.69	80.31	43.14	28.03	1.87	15.34
G299	ETSL101512	10.07	69.09	19.66	80.34	19.22	26.81	1.79	15.82
G300	ETSL101515	9.63	68.65	19.58	80.42	27.35	26.6	1.49	15.63
G301	ETSL101523	9.59	68.78	19.83	80.17	28.56	23.61	1.28	15.53
G302	ETSL101524	9.22	68.89	19.74	80.26	37.95	29.52	1.81	14.98
G303	ETSL101527	9.44	68.98	19.73	80.27	40.21	27.04	1.69	15.31
G304	ETSL101535	9.26	68.44	19.51	80.5	17.68	25.82	1.6	15.24
G305	ETSL101536	10.32	68.67	19.53	80.47	20.96	26.5	1.92	15.55
G306	ETSL101540	9.42	68.95	19.71	80.29	33.22	25.07	1.5	15.36
G307	ETSL101550	9.28	68.85	19.68	80.32	39.39	27.24	1.6	15.49
G308	ETSL101555	10.22	68.67	19.87	80.14	27.36	25.41	1.61	15.33
G309	ETSL101556	9.68	68.87	19.7	80.3	45.83	27.31	1.81	15.48
G310	ETSL101559	10.37	68.4	19.69	80.31	39.22	27.72	1.6	15.7
G311	ETSL101561	9.85	68.94	19.83	80.17	31.75	24.97	1.69	15.25
G312	ETSL101563	10.39	68.02	19.81	80.19	37.14	28.62	1.62	15
G313	ETSL101564	9.69	68.93	19.66	80.34	23.3	24.88	1.29	15.62
G314	ETSL101568	10.22	68.85	19.5	80.5	19.05	25.43	1.7	15.97
G315	ETSL101571	10.2	69.04	19.62	80.38	23.99	25.63	1.78	15.6
G316	ETSL101578	10.09	68.6	19.54	80.46	31.76	26.09	1.67	15.6
G317	ETSL101586	9.29	68.51	19.41	80.59	33.36	27.43	1.77	15.51
G318	ETSL101629	10.29	68.42	19.41	80.59	18.25	26.59	1.93	15.98
G319	ETSL101640	9.16	69.15	19.99	80.01	18.15	25.54	1.52	15.29
G320	ETSL101643	9.45	68.88	19.72	80.28	50.6	27.23	1.96	15.32
G321	ETSL101654	9.49	68.72	19.43	80.57	37.34	30.55	1.91	15.53
G322	ETSL101671	9.16	68.71	19.47	80.53	49.7	33.19	2.23	15.5
G323	ETSL101673	9.42	69	19.66	80.35	19.72	26.17	2.03	15.44
G324	ETSL101676	10.13	68.55	19.52	80.48	20.69	26.7	1.89	15.47
G325	ETSL101681	9.04	69.21	19.87	80.13	22.13	26.01	1.49	15.22
G326	ETSL101686	9.39	69.01	19.67	80.33	24.67	25.51	1.8	15.55
G327	ETSL101687	8.85	69.18	19.62	80.38	34.4	27.14	1.71	15.62
G328	ETSL101698	10.03	68.9	19.69	80.31	32.38	25.58	1.7	15.4
G329	ETSL101704	8.97	69.03	19.56	80.44	43.11	29.86	1.93	15.51
G330	ETSL101712	8.71	69.01	19.74	80.26	44.5	33.06	1.83	15.11

G331	ETSL101715	9.84	68.71	19.75	80.25	20.63	26.44	1.5	15.23
G332	ETSL101719	9.47	68.98	19.6	80.4	23.65	26.54	1.79	15.65
G333	ETSL101722	9.94	68.47	19.59	80.41	21.15	27.98	2.09	15.44
G334	ETSL101723	9.39	68.62	19.42	80.58	24.67	27.96	1.54	15.78
G335	ETSL101725	10.07	68.73	19.69	80.31	36.38	24.28	1.59	15.52
G336	ETSL101730	9.34	68.73	19.63	80.37	41.76	26.59	1.65	15.49
G337	ETSL101732	8.5	68.96	19.72	80.28	53.29	34.99	1.91	15.52
G338	ETSL101749	10.05	68.6	19.64	80.36	44.79	26.73	1.76	15.52
G339	ETSL101760	9.12	68.82	19.78	80.22	19.2	24.1	1.59	15.48
G340	ETSL101766	10.62	68.82	19.55	80.45	41.88	25.66	1.92	15.54
G341	ETSL101848	9.8	68.14	19.07	80.93	31.66	31.78	2.36	15.52
G342	ETSL101851	10.32	68.77	19.49	80.51	19.78	27.88	1.81	15.89
G343	ETSL101856	9.82	68.98	19.57	80.43	22.19	27.29	1.92	15.27
G344	ETSL101858	9.73	68.87	19.51	80.49	29.08	25.89	1.69	15.45
G345	ETSL101868	9.77	68.41	19.5	80.5	24.08	26.78	1.73	15.42
G346	IS25531	8.28	68.58	19.82	80.19	49.72	31.04	1.92	15.83
G347	IS38254	9.65	68.91	19.45	80.55	27.16	28.99	2.07	15.71
G348	IS38255	10.45	68.54	19.41	80.59	23.86	24.97	1.61	15.87
G349	IS38259	8.95	69.21	19.34	80.66	25.12	27.5	1.92	15.32
G350	IS38275	9.56	68.74	19.6	80.4	32.93	28.06	1.81	15.5
G351	IS38279	9.1	69.03	19.61	80.39	41.59	26.94	1.89	15.56
G352	IS38290	9.56	68.74	19.6	80.4	32.93	28.06	1.81	15.5
G353	IS38303	8.61	68.81	19.45	80.55	38.68	33.9	2.12	15.56
G354	IS38306	9.31	68.75	19.71	80.29	33.83	27.32	1.65	15.36
G355	IS38319	9.15	67.44	19.33	80.67	44.46	37.53	2.3	16.24
G356	IS38326	10.09	68.22	19.23	80.77	29.38	27.02	1.77	15.57
G357	IS38331	8.63	68.84	19.37	80.63	21.87	28.01	2.1	15.88
G358	IS38353	11.11	67.99	19.47	80.53	24.17	28.02	1.96	15.97
G359	IS38412	9.12	68.74	19.45	80.55	34.14	28.07	1.68	15.57
G360	P9830	10.51	68.49	19.52	80.48	18.46	27.09	1.55	15.51
G361	PML981446	9.43	69.05	19.86	80.14	54.57	29.21	2.04	15.48
G362	PML981474	10.52	67.9	19.36	80.64	34.3	31.98	2.07	15.53
G363	PML981476	9.07	69.3	19.7	80.29	34.74	24.12	1.66	15.46
G364	PML981488	9.33	68.83	19.79	80.21	29.79	25.67	1.47	15.49
G365	TAM428	9.98	68.62	19.66	80.34	19.26	25.56	1.58	15.5
	**Mean**	**9.56**	**68.74**	**19.6**	**80.4**	**32.93**	**28.05**	**1.81**	**15.5**

AM=Amylose, AMP=Amylopectin, MST=Moisture
