# Supplementary material for: Kernel composition in sorghum landraces revealed via analyses of genotype-by-environment interactions
Source: PLoS One. 2025 Apr 7;20(4):e0320513. doi: 10.1371/journal.pone.0320513 (PMC11975078; doi:10.1371/journal.pone.0320513)
Supplement: Attachment — Submitted filename: Responses to Reviewers.docx [file pone.0320513.s005.docx]

**A POINT-BY-POINT LIST OF RESPONSES TO THE REVIEWERS’ COMMENTS:**

**Responses to Reviewer #1:**

**Abstract:**

**Comment:**

1. *Include additional sentences at the end of the abstract to explain how landraces with high protein and zinc content, which exhibit stability across environments, can be utilized in both plant breeding and human nutrition.*
   **Response:**

**We have revised the abstract and included the stable, high-nutritional-value landraces in common for traits [lines 18-25].**

2. *Clarify the aims in the abstract.*
   **Response: The aims have been clarified as suggested, emphasizing the impact of diverse genotypes and growing locations on grain quality [lines 26-28].**

**Introduction:**
 3. *Incorporate references to recent studies (e.g., Kumar et al., 2023; Madhusudhana et al., 2023) and explain what sets this study apart.*
**Response: We have added the suggested references and highlighted how our study builds on these works [lines 38-53].**

4. *Replace outdated references with updated ones.*
   **Response:** Outdated references have been updated with more recent and relevant citations.
5. *Include high-quality citations in the literature review.*
   **Response: The recommended citations have been incorporated to enrich the discussion and introduction, particularly regarding G×E interaction analyses.**
6. *Remove parenthesis from line 55 and add the missing bracket on line 56.*
   **Response: The editorial errors have been corrected.**

**Materials and Methods (M&M):**
7. *Clarify the rationale for different planting dates in the two years.*
**Response:** An explanation of the different planting dates has been included, addressing environmental conditions and logistical constraints.

8. *Remove redundant use of "landrace" in line 105.*
   **Response:** The redundancy has been eliminated.
9. *Clarify the total number of landraces (361 or 358).*
   **Response: The correct number (361 landraces and 4 checks) has been clarified and consistently reflected throughout the manuscript [lines 113-121, Table 4].**
10. *Indicate the method used for analyzing grain quality and eliminate superfluous sentences in line 123.*
    **Response:** The methodology for grain quality analysis has been explicitly described, and unnecessary text has been removed.

**Results:**
11. *Include the results of the combined analysis of variance (c-ANOVA).*
**Response: The c-ANOVA results have been added to the manuscript [Table 5].**

12. *Provide units for quality traits in Table 6.*
    **Response: Units have been included for each trait in Table 6.**
13. *Correct the genotype number in line 166 [*“G2649”]*.*
    **Response: The number G264 has been corrected.**
14. *Identify genotypes with high nutritional value.*
    **Response: We have included a description of genotypes with high starch, zinc, iron, and protein levels in the results section.**
15. *Ensure figures (2, 3, 4, etc.) are referenced and properly numbered.*
    **Response: The figures have been reviewed, correctly referenced, and consistently numbered.**
16. *Ensure consistency in reporting the number of sorghum landraces (361, 364, or 358).*
    **Response: We have ensured consistency and confirmed the number as 361 landraces and 4 checks throughout the manuscript [lines 113-120, Table 4].**
17. *Include missing figures (8, 9, and 10) in the manuscript.*
    **Response: The missing figures have been included and numbered correctly.**
18. *Revise the paragraph on genotype performance and stability.*
    **Response: The paragraph has been rewritten to enhance readability and provide a more dynamic analysis of genotype performance.**
19. *Perform correlation analysis for grain quality traits.*
    **Response: A correlation analysis has been performed and included in a new section titled "Association Between Traits" and Table 7 (Lines 459-469)**

**Discussion:**
21. *Avoid reiterating results and highlight genotypes with high nutritional value and stability.*
**Response: The discussion has been revised to focus on the implications of stable genotypes with high nutritional value for breeding programs.**

22. *Calculate heritability and discuss its implications.*
    **Response: Heritability estimates have been calculated and discussed in the context of selecting genotypes for enhanced micronutrient levels.**

**Responses to Reviewer #2:**

1. *Perform Bartlett’s test for combined analysis.*
   **Response: Bartlett’s test has been conducted.**
2. *Include the combined analysis of variance table.*
   **Response: The c-ANOVA table has been added (Table 5).**
3. *Describe how variance components were calculated.*
   **Response: A detailed explanation of variance component calculations has been added to the Materials and Methods section.**
4. *Correct figure numbering.*
   **Response: Figure numbering has been reviewed and corrected throughout.**
5. *Clarify superior genotypes in the ranking bi-plot.*
   **Response: Superior genotypes have been clearly identified and highlighted in the ranking biplot.**
6. *Address how results with differing superior genotypes for traits can be used.*
   **Response: A discussion has been included on how differing superior genotypes for traits can guide targeted breeding strategies for specific environments and nutritional goals.**
