# Supplementary material for: Kernel composition in sorghum landraces revealed via analyses of genotype-by-environment interactions
Source: PLoS One. 2025 Apr 7;20(4):e0320513. doi: 10.1371/journal.pone.0320513 (PMC11975078; doi:10.1371/journal.pone.0320513)
Supplement: Attachment — Submitted filename: PONE review_30.10.2024.docx [file pone.0320513.s002.docx]

**Manuscript Number:** PONE-D-24-44523
**Manuscript Title:** Kernel compositions in sorghum landraces revealed via the analyses of genotype-by environment Interactions

**Reviewer:**

Dear authors,

I carefully read the MS entitled " Kernel compositions in sorghum landraces revealed via the analyses of genotype-by environment interactions". In this paper, diverse genotypes and various growing locations impact was shown on the chemical and physical properties of 361 sorghum grain. GGE ranking biplots identified genotypes with least G × E and genotypes identified for their superior nutritional. While the paper is intriguing, I have some significant comments and suggestions that need to be addressed before accepting it.

**Abstract:**

- Include additional sentences at the end of the abstract that explain how landraces with high protein and zinc content, which exhibit stability across various environments, can be utilized in both plant breeding and human nutrition.

- Please clarify the aims in the abstract. For example, to assess how diverse genotypes and various growing locations impact the chemical and physical properties of sorghum grain, we evaluated 361 sorghum landraces over two growing seasons.

**Introduction:**

- What sets this study apart from recent research, such as the one conducted by Kumar et al. (2023) titled “Exploiting Indian landraces to develop biofortified grain sorghum with high protein and minerals” and study by Madhusudhana et al. (2023) titled “Genetic variability, G× E interaction and stability for iron and zinc content in sorghum grains in advanced breeding lines”? Please incorporate these references into both the introduction and discussion sections of the paper.

- Please replace outdated references with updated ones.

- I recommend including several high-quality citations in the literature review of the manuscript. These citations can be used to characterize environments and distinguish stable genotypes from unstable genotypes in various crops during the discussion section. The following citations are suggested:

Hassani, M., Heidari, B., Dadkhodaie, A., & Stevanato, P. (2018). Genotype by environment interaction components underlying variations in root, sugar and white sugar yield in sugar beet (Beta vulgaris L.). *Euphytica*, *214*, 1-21.

Shahriari, Z., Heidari, B., & Dadkhodaie, A. (2018). Dissection of genotype× environment interactions for mucilage and seed yield in Plantago species: Application of AMMI and GGE biplot analyses. *PloS one*, *13*(5), e0196095.

- Remove the parenthesis from line 55.

- A bracket is missing on line 56.

**M&M:**

- Why was the planting date different in the first and second year?

- At line 105, the term "landrace" was repeated. Please remove the redundant use of "landrace."

- How many landraces were evaluated in the current study - 361 or 358? Please provide clarification.

- In line 123, the authors should clearly indicate the method used for analyzing grain quality in sorghum and eliminate any superfluous sentences.

**Results:**

- The manuscript does not display the results of the combined analysis of variance (c-ANOVA). Please include the c-ANOVA results in the manuscript.

- Please include the unit for each quality trait in Table 4.

- Line 166, the number of genotypes “G2649” should be corrected.

-Finding a landrace with high nutritional value is crucial, particularly in terms of iron, zinc, and protein content. Has a genotype been identified in the current study that exhibits elevated levels of zinc, iron, and protein?"

- In the manuscript, Figures 2, 3, and 4 were not referenced. The authors should review the figures and ensure they are correctly referenced in the results section.

-The manuscript reports varying numbers of sorghum landraces, with some places listing 361, while others list 364 or 358. It is important to ensure consistency throughout the entire manuscript.

- It would be better to use moisture-stress conditions throughout the entire manuscript, rather than stress conditions.

- In manuscript figures 8, 9, and 10 was cited while until figure 7 was shown in results. Please checked and missed figures should be shown in the manuscript.

-The paragraph titled "Genotype Performance and Stability" needs to be revised to enhance its readability and engagement. The current writing style is lackluster and should be elevated to provide a more dynamic analysis of the topic, focusing on both the highest and lowest performing genotypes.

- I suggest performing correlation analysis for grain quality traits, such as protein, iron, and zinc concentration. These results could be included in a separate section of the manuscript titled "Association Between Traits." Understanding the relationships between traits is crucial for developing an effective breeding strategy for genetic enhancement. By examining correlations between micronutrients and rank correlations across different locations, breeders can better allocate their resources for indirect selection approaches. This will ultimately lead to improvements in both micronutrients by testing in the minimum required target test environments.

**Discussion**

- It is generally recommended to refrain from reiterating the results in the discussion section. Instead, authors should consider incorporating a dedicated paragraph in the discussion to highlight genotypes that exhibit high nutritional value and stability across various environments. These particular genotypes hold promise for inclusion in breeding programs aimed at enhancing nutritional content.
- In line 437, the author discusses the high GEI for Fe and Zn content, suggesting that their heritability may be a contributing factor. Although the authors did not calculate heritability. I recommend that the author calculated heritability, as high heritability within a set of genotypes suggests a strong potential for selecting genotypes to enhance micronutrient levels through effective breeding strategies.
