# Supplementary material for: Kernel composition in sorghum landraces revealed via analyses of genotype-by-environment interactions
Source: PLoS One. 2025 Apr 7;20(4):e0320513. doi: 10.1371/journal.pone.0320513 (PMC11975078; doi:10.1371/journal.pone.0320513)
Supplement: Attachment — Submitted filename: Response to Editor Comments.docx [file pone.0320513.s006.docx]

**Response to Editor Comments:**

1. **Comment: Specify the name of the experimental design used for ANOVA.**
   **Response:** Thank you for your suggestion. We have now explicitly stated the experimental design a partially replicated (p-rep) design was used in a row-column arrangement in across environments (Lines 119-120).
2. **Comment: Specify components of phenotypic variances used in heritability formula.**
   **Response:** We appreciate this observation. We have revised the manuscript to clearly define the components used in the heritability formula in the Materials and Methods section (Lines 166-169).
3. **Comment: Define all abbreviations in the text and tables.**
   **Response:** We acknowledge the oversight and we have also carefully checked and ensured that all other abbreviations are properly defined throughout the manuscript.
